# Therapeutic Cancer Vaccination With a Peptide Derived From the Calreticulin Exon 9 Mutations Induces Strong Cellular Immune Responses in Patients With *CALR*-Mutant Chronic Myeloproliferative Neoplasms

**DOI:** 10.3389/fonc.2021.637420

**Published:** 2021-02-26

**Authors:** Jacob Handlos Grauslund, Morten Orebo Holmström, Nicolai Grønne Jørgensen, Uffe Klausen, Stine Emilie Weis-Banke, Daniel El Fassi, Claudia Schöllkopf, Mette Borg Clausen, Lise Mette Rahbek Gjerdrum, Marie Fredslund Breinholt, Julie Westerlin Kjeldsen, Morten Hansen, Steffen Koschmieder, Nicolas Chatain, Guy Wayne Novotny, Jesper Petersen, Lasse Kjær, Vibe Skov, Özcan Met, Inge Marie Svane, Hans Carl Hasselbalch, Mads Hald Andersen

**Affiliations:** ^1^ National Center for Cancer Immune Therapy (CCIT-DK), Department of Oncology, Copenhagen University Hospital, Herlev, Denmark; ^2^ Department of Hematology, Copenhagen University Hospital, Herlev, Denmark; ^3^ Department of Medicine, Copenhagen University, Copenhagen, Denmark; ^4^ Department of Pathology, Zealand University Hospital, Roskilde, Denmark; ^5^ Department of Pathology, Copenhagen University Hospital, Herlev, Denmark; ^6^ Department of Hematology, Oncology, Hemostaseology and Stem Cell Transplantation, Faculty of Medicine, RWTH Aachen University, Aachen, Germany; ^7^ Department of Hematology, Zealand University Hospital, Roskilde, Denmark; ^8^ Institute for Immunology and Microbiology, Copenhagen University, Copenhagen, Denmark

**Keywords:** myeloproliferative neoplasms, cancer immune therapy, cancer vaccines, neo-antigen, calreticulin

## Abstract

**Background:**

The calreticulin (*CALR*) exon 9 mutations that are identified in 20% of patients with Philadelphia chromosome negative chronic myeloproliferative neoplasms (MPN) generate immunogenic antigens. Thus, therapeutic cancer vaccination against mutant CALR could be a new treatment modality in *CALR*-mutant MPN.

**Methods:**

The safety and efficacy of vaccination with the peptide CALRLong36 derived from the *CALR* exon 9 mutations was tested in a phase I clinical vaccination trial with montanide as adjuvant. Ten patients with *CALR*mut MPN were included in the trial and received 15 vaccines over the course of one year. The primary end point was evaluation of safety and toxicity of the vaccine. Secondary endpoint was assessment of the immune response to the vaccination epitope (www.clinicaltrials.gov identifier NCT03566446).

**Results:**

Patients had a median age of 59.5 years and a median disease duration of 6.5 years. All patients received the intended 15 vaccines, and the vaccines were deemed safe and tolerable as only two grade three AE were detected, and none of these were considered to be related to the vaccine. A decline in platelet counts relative to the platelets counts at baseline was detected during the first 100 days, however this did not translate into neither a clinical nor a molecular response in any of the patients. Immunomonitoring revealed that four of 10 patients had an *in vitro* interferon (IFN)-γ ELISPOT response to the CALRLong36 peptide at baseline, and four additional patients displayed a response in ELISPOT upon receiving three or more vaccines. The amplitude of the immune response increased during the entire vaccination schedule for patients with essential thrombocythemia. In contrast, the immune response in patients with primary myelofibrosis did not increase after three vaccines.

**Conclusion:**

Therapeutic cancer vaccination with peptide vaccines derived from mutant *CALR* with montanide as an adjuvant, is safe and tolerable. The vaccines did not induce any clinical responses. However, the majority of patients displayed a marked T-cell response to the vaccine upon completion of the trial. This suggests that vaccines directed against mutant CALR may be used with other cancer therapeutic modalities to enhance the anti-tumor immune response.

## Introduction

The Philadelphia chromosome-negative chronic myeloproliferative neoplasms (MPN) are chronic blood cancers originating in the hematopoietic stem cells in the bone marrow (1, 2). MPN have overlapping signs and symptoms and thus may be difficult to distinguish from one another. Essential thrombocythemia (ET) and polycythemia vera (PV) are the non-advanced MPN, with patients presenting elevated levels of peripheral blood cells, whereas primary myelofibrosis is the advanced disease characterized by bone marrow fibrosis, and patients often present with cytopenia. Prefibrotic or early myelofibrosis (PreMF) was recently acknowledged as a distinct MPN with patients presenting with early signs of myelofibrosis (3). Allogeneic hematopoietic stem cell transplantation (alloHSCT) remains the only curative treatment for MPN, and as patients with ET and PV may live for several decades (4, 5) alloHSCT, which has a high treatment-related mortality, is not used for the treatment of these diseases. AlloHSCT is used for younger and fit patients with PMF (6), but as the majority of patients with PMF are of older age, alloHSCT is not used routinely for these patients. Due to the elevated blood cell counts and the presence of the MPN-driver mutations per se, patients with ET and PV have a dramatically increased risk of thromboembolic episodes and hemorrhage. Hence, the treatment for ET and PV aims to lower this risk with cytoreductive therapy (7) such as hydroxyurea (HU), anagrelide (ANA), and pegylated interferon alpha (IFN-α). HU is a weak chemotherapeutic agent that reduces peripheral blood cell counts, whereas ANA is solely used to reduce the platelet count. IFN-α is a cytokine that in the past has been used to treat several cancers, such as multiple myeloma, chronic myelogenous leukemia (CML), malignant melanoma and renal cancer (8) but in general with a low-rate of success except partly for CML (9). In MPN, IFN-α can induce major molecular remissions, complete hematological responses (10, 11), and even normalization of bone marrow architecture in some patients (11). As IFN-α is an immunostimulatory cytokine, it has been speculated that one of its mechanisms of action is to normalize the immune environment in patients with MPN (12, 13).

Several lines of evidence show that the immune system is severely dysregulated in MPN (13–15). Cytokine levels and the immune phenotype in patients differ from those in healthy donors. This immune derangement is likely partially driven by the driver mutations identified in MPN, as they all affect the JAK-STAT signaling pathway which is of paramount importance in the regulation of the immune-system (16). The mutational landscape in MPN is highly homogeneous, as approximately 90% of patients harbor a mutation in the Janus kinase-2 (*JAK2*), myeloproliferative leukemia virus (*MPL*), or calreticulin (*CALR*) gene. The *JAK2*V617F mutation has been identified in 98% of patients with PV and in 50% of patients with ET and PMF (17, 18). The *MPL* mutations are identified in 5–10% of patients with ET and PMF (19), and the *CALR* exon 9 mutations are found in 20–25% of patients with ET and PMF (20, 21). Whereas the *JAK2* and *MPL* mutations generate single-amino-acid substitutions in their respective proteins, the *CALR* deletion or insertion mutations result in frameshift mutations that generate a novel mutant C-terminus different from the wild-type (wt) CALR C-terminus (20, 21). Interestingly, peptides derived from the mutant C-terminus are recognized by peripheral blood mononuclear cells (PBMCs) isolated both from patients with *CALR*-mutant (*CALR*mut) MPN and from healthy donors (22, 23), and T cells isolated from patients with *CALR*mut MPN recognize and kill autologous *CALR*mut cells in a *CALR*mut-dependent manner (22). These data have established that the *CALR* mutations encode tumor-specific antigens (TSAs) that are recognized by patient T cells.

Studies on other TSAs have shown that they may be targeted by the immune system (24), and clinical trials have used this in the setting of therapeutic cancer vaccination, where vaccination with peptides derived from the TSA is aimed at inducing/enhancing the tumor-specific immune response (25). The first TSAs targeted by therapeutic cancer vaccines arose from *RAS* mutations, which are the most common somatic mutations in human cancer (26). Initial studies showed that patient T cells respond to stimulation *in vitro* with epitopes derived from mutant RAS (27, 28), and that these T cells can kill HLA-matched *RAS*-mutant cancer cells (29). These results spurred the first therapeutic cancer vaccines using TSA-derived peptides, where vaccination against mutant *KRAS* induced or enhanced an immune response specific to the *KRAS* mutation-derived TSA (30). Several clinical vaccination trials testing vaccination against mutant *KRAS* have shown a survival benefit to patients who develop an immune response to the vaccination antigen (31–33), thus establishing the potential of TSA-specific therapeutic cancer vaccines.

Given the high immunogenicity of the *CALR*mut-derived TSA, we initiated a phase I clinical vaccination trial with a CALRmut-derived peptide in 10 patients with *CALR*mut MPN. Safety and tolerability were the primary end points, attainment of immune response to the CALRmut TSA was the secondary end point, and clinical response was the tertiary end point. The vaccine was safe and tolerable, and although the vaccines did not induce any clinical responses, they induced marked alterations in the immune phenotype and immune response to the vaccination antigen.

## Subject, Materials and Methods

### Study Design

This was a phase I clinical vaccination trial testing the safety and immunogenicity of vaccination with the CALRLong36 peptide with montanide as an adjuvant. Patients from the Departments of Hematology at Herlev and Gentofte Hospital, Copenhagen, Denmark, as well as Zealand University Hospital, Roskilde, Denmark were enrolled in the trial between August 30^th^, 2018 and February 17^th^ 2019 with the last vaccination administered January 14^th^ 2020. The study was conducted in accordance with the Helsinki Declaration, and Good Clinical Practice recommendations. All participants provided written informed consent before enrollment in the trial. The protocol was approved by the Ethics Committee of the Zealand Region, the National Board of Health, and the Danish Data Protection Agency, and registered at www.clinicaltrials.gov (NCT03566446; date of registration June 25th, 2018). One patient from whom we wished to isolate PBMC after the end of the study provided informed consent for this in accordance with an approval from the Ethics Committee of Zealand Region (approval number SJ-456).

As therapeutic cancer vaccines have not been tested earlier in patients with MPN, we had no basis for any power calculations. Based on our previous experience with phase I clinical vaccination trials, we chose to include 10 patients in the trial. Main inclusion criteria were a diagnosis of ET, PreMF, or PMF according to the WHO criteria, verified mutation in exon 9 of *CALR*, and Eastern Cooperative Oncology Group performance status of ≤2. A full list of the inclusion and exclusion criteria can be found in **Supplementary Figure 1**. Patients were allowed concurrent treatment with IFN-α, HU, or ANA in any combination, but no other anti-neoplastic or anti-MPN treatments were allowed.

### Vaccine Composition and Treatment Schedule

Each vaccine was composed of 200 µg CALRLong36, a 36-amino acid peptide (RMRRMRRTRRKMRRKMSPARPRTSCREACLQGWTEA) spanning the entire mutant C-terminus created by the most frequent *CALR* mutations. The peptide was provided by Polypeptide (Strasbourg, France). The peptide was dissolved in 500 µL sterile water and mixed with 500 µL montanide ISA-51 just prior to administration. The vaccine was administered subcutaneously, and patients were vaccinated with a total of 15 doses, with the first six doses administered every second week and the final nine doses every fourth week. A Gant chart of the vaccination schedule is provided in **Supplementary Figure 2**.

### Evaluation of Adverse Events and Clinical Response

Adverse events (AE) were assessed according to the Common Terminology Criteria for Adverse Events (CTCAE) version 4.03. Before receiving the first vaccination, each patient underwent a full medical examination and had an electrocardiogram recorded. This was repeated before each of the first three vaccinations, and then before the 7^th^ and after the 15^th^ vaccination. Biochemical tests on peripheral blood were performed before each vaccination. The patients underwent a full bone marrow examination and analysis by next-generation sequencing (NGS) performed on peripheral blood prior to the first vaccination and at end of study. The clinical response was evaluated according to the response criteria described by the European LeukemiaNet (34, 35).

### Evaluation of the *CALR*mut Allelic Burden and Next-Generation Sequencing

PCR detection of *CALR* exon 9 frameshift mutations was performed as described by Klampfl et al. (20). Primers used are: CALR Forward: 5′-Fam-GGCAAGGCCCTGAGGTGT-3′, CALR Reverse: 5′-GGCCTCAGTCCAGCCCTG-3′. The PCR product was run on a ABI3500 Genetic Analyzer (Thermo Fisher Scientific) and mutated CALR allele burden calculated as 100*AUC (area under the curve) for mutated CALR/(AUC for mutated + AUC wildtype).

Next generation sequencing was performed on a custom made panel of 42 genes designed to capture mutations implicated in myeloid malignancies (*ASXL1, BCOR, BRAF, CALR, CEBPA, CBL, CSF3R, CUX1, DNMT3A, ETV6, EZH2, FLT3, GATA2, GNAS, IDH1, IDH2, JAK2, KIT, KRAS, MLL3, MPL, MYD88, NF1, NFE2, NOTCH1, NPM1, NRAS, PHF6, PPM1D, PTPN11, RB1, RIT, RUNX1, SETBP1, SF3B1, SH2B3, SRSF2, STAG2, TET2, TP53, U2AF1, ZRSR2*). Genomic DNA was purified from peripheral blood at baseline and 12 months after the first vaccination of 10 *CALR* mutated MPN patients. Libraries were prepared using the Nextera Flex for Enrichment protocol (Illumina^®^ Inc, San Diego, CA, USA), and 2 × 150 bp paired-end sequencing was done on the NextSeq 500 platform (Illumina^®^ Inc, San Diego, CA, USA). Quality control of all sequencing runs was assessed using the Illumina Sequencing Analysis Viewer (SAV) software. CLC Genomics Workbench v.12.0.3 software was applied to align sequencing data to the human reference genome (GRCh37/hg19) and to call variants from the mapped reads. Annotation and filtering of variants were performed using the VarSeq™ v.2.2.1 software (Golden Helix, Inc., Bozeman, MT, USA). Variants with coverage <100x, variant allele frequency (VAF) <1%, introns, germline, and SNPs with minor allele frequency >1% (ExAC variant frequencies, Broad Institute, MA, USA) were excluded. A mutation with VAF <1% in either a baseline or treatment sample was retained if the mutation had a VAF >1% in the paired sample. In addition, only variants classified as either pathogenic or likely pathogenic were retained for further analysis.

### Isolation of Bone Marrow and Peripheral Blood Mononuclear Cells

Blood samples for isolation of PBMCs were obtained at baseline, after three vaccinations, after six vaccinations, and after 15 vaccinations. Samples were kept at room temperature (RT) for ≤5 h until handling. PBMCs were isolated by gradient centrifugation of heparinized blood on Lymphoprep (STEMCELL Technologies) in LeucoSep tubes (Greiner Bio-One). Isolated PBMCs were cryopreserved in 90% human serum (Sigma-Aldrich) with 10% DMSO (Sigma-Aldrich), using controlled-rate freezing (Cool-Cell, Biocision) in a −80°C freezer. The next day, the ampoules were transferred to −140°C.

Heparinized bone marrow samples (10 mL in a heparinized tube) were obtained at baseline and after 15 vaccinations. Ortho-Lysing Buffer diluted 10× in H_2_0 was added to half of the sample, followed by centrifugation and incubation for 15 min in the dark. The other half of the sample was handled and cryopreserved following the same procedure as for PBMCs.

### Delayed-Type Hypersensitivity and Skin-Infiltrating Lymphocytes

Presence of tumor-specific T cells in biopsies from delayed-type hypersensitivity (DTH) testing post-vaccination is correlated with clinical outcome (36). We assessed the presence of vaccine-reactive cells at DTH sites after six vaccinations. On the lower back, we performed three intradermal injections of CALRLong36 without adjuvant and one control injection of sterile water, without peptide. At 48 h post-DTH injection, skin reaction was measured and punch biopsies were taken from the sites of CALRLong36-containing injections and cut into fragments. Fragments were cultured in 24-well plates for 3–5 weeks in RPMI-1640 with 10% human serum and 100 U/mL or 6,000 U/mL interleukin-2 (IL-2) with penicillin, streptomycin, and fungizone. Three times weekly, half the medium was replaced with fresh medium containing IL-2. Skin-infiltrating lymphocytes (SKILs) emigrated from the biopsies. After 3–5 weeks, SKILs were harvested and tested in ELISPOT assays (see below). The remaining SKILs were cryopreserved, as described for PBMCs.

### IFN-γ ELISPOT Assay

In order to assess the immune response against CALRLong36, we used IFN-γ ELISPOT assays performed on PBMCs as described in detail earlier (37). In short, PBMCs were thawed and stimulated with CALRLong36 and then stimulated with IL-2 (120 U/mL) the next day. The cells were incubated for 14 days in x-vivo (Lonza, Belgium) supplemented with 5% human serum before restimulation with CALRLong36 in the ELISPOT wells with unstimulated wells as negative controls. The ELISPOT plates were analyzed using the ImmunoSpot Series 2.0 Analyzer (CTL, Shaker Heights, Ohio). ELISPOT assays were performed in triplicate experiments with a concentration of 2.5–3.5 × 10^5^ cells/well. For graphical representation the spot counts were normalized to 3 × 10^5^ cells/well. The mean spot count per experiment was calculated by subtracting the mean spot count of the negative control wells from the mean spot count in the peptide-stimulated wells. ELISPOT assays on SKILs were run in triplicates or quadruplicates with 3 × 10^5^ cells/well.

### Intracellular Cytokine Staining

In order to identify the phenotype of cells releasing IFN-γ and TNF-α upon stimulation with CALRLong36 we used intracellular cytokine staining on *in vitro*-stimulated PBMC. The *in vitro* stimulation followed the same steps as for ELISPOT analysis and the stimulation, staining, and flow cytometric analysis of PBMCs followed our previously described method (37).

### Phenotyping of Peripheral Blood Mononuclear Cells Using Fluorescence-Activated Cell Sorting (FACS)

Cryopreserved PBMCs from different timepoints were thawed and washed in preheated PBS. The Fc-receptors were blocked by incubation with human IgG (human IgG, 50 µg/ml), and dead cells were stained using Fixable Near-IR Dead cell stain kit (Thermo-Fisher) and incubated at 4°C for 10 min. Next, cells were stained by fluorochrome labelled antibodies and stained in the dark at 4°C for 20 min. After staining, the cells were washed and acquired using a NovoCyte Quanteon Flow Cytometer (Agilent, Santa Clara, CA). Gating strategy is provided in **Supplementary Figure 3** and a list of antibodies used in **Supplementary Figure 4**. Data were analyzed using the NovoExpress 1.4.1 software. All gates at baseline were applied to all timepoints. Illustration of data were performed using Graphpad Prism v 8.0 (GraphPad Software. Inc.).

### Statistical Analysis

ELISPOT responses were analyzed using the distribution-free resampling (DFR) method (38). Differences in immune subset populations between different time points were analyzed using the Wilcoxon matched-pairs signed-rank test. As this exploratory analysis was descriptive and done *post hoc*, no formal multiple testing corrections were performed. *p* values ≤ 0.05 were considered significant. All analyses were performed in Graphpad Prism v 8.0 (GraphPad Software. Inc.), apart from the DFR analyses which were performed using the statistical analysis program R.

## Results

### Patient Characteristics

Eleven patients were included and evaluated by examination of the bone marrow biopsies and blood samples prior to initiation of vaccinations. Due to compliance issues, one patient was excluded, and thus 10 patients continued in the trial. All patients received the planned 15 vaccine doses over the course of one year. The male:female ratio was 5:5, the median age at inclusion was 59.5 years (range 41-73 years), and the median duration of disease was 6.5 years (range 2–26 years) (**Tables 1** and **2**). At study entry, four patients had ET, three patients PMF, two patients post-essential thrombocythemia MF (PET-MF) and one patient prefibrotic/early myelofibrosis (PreMF). All but one patient had received MPN-directed therapy including HU, ANA, or INF-α prior to enrollment. Of the nine treated patients, eight were treated with INF-α and one with ANA at the start of vaccination. This one patient treated with ANA had received IFN-α earlier, however IFN-α had been withdrawn due to side effects. Median platelet count was 283 × 10^9^/l (range 145 × 10^9^/l–728 × 10^9^/l), median hemoglobin count was 7.95 mmol/l (range 6.5 mmol/l–9.2 mmol/l), and median leucocyte count was 4.9 × 10^9^/l (range 3.3 × 10^9^/l–10.5 × 10^9^/l). Four patients presented with splenomegaly on clinical examination. Four patients had the type 1 CALR-mutation, one patient a type-1-like CALR-mutation and five had the type 2 CALR-mutation. The median *CALR*mut VAF was 40.5% (range, 1–49%).

**Table 1:** Patient characteristics.

Sex	Female n= 5, Male n=5
Age at inclusion in years, median (min–max)	59.5 (41-73)
Duration of disease in years, median (min–max)	6.5 (2-26)
Diagnosis	ET n=4 PMF N=3 PreMF n=1 PET-MF n=2
Treatment	Pegylated Interferon-alpha n=8 Anagrelide n=1 No Treatment n=1
Platelet count at inclusion, median (min-max)	283x10^9^/l (145x10^9^/l–728x10^9^/l)
Hemoglobin at inclusion, median (min-max)	7.95 mmol/l (6.5mmol/l–9.2mmol/l)
Leukocyte count at inclusion, median (min-max)	4.9x10^9^/l (3.3x10^9^/l–10.5x10^9^/l)
Splenomegaly at inclusion	n=4
CALR-mutation type	Type 1 (52 bp del) n=5 Type 2 (5 bp ins) n=5
% CALRmut VAF at inclusion, median (min-max)	40.5% (1% - 49%)

**Table 2:** Individual patient data for all patients in the trial.

Patient number	1	2	3	4	5	6	7	8	9	10
Age (years)	73	70	61	61	43	52	41	57	67	58
Sex	Female	Female	Male	Female	Male	Male	Male	Female	Female	Male
Duration of disease (years)	7	18	26	2	22	3	4	14	3	6
Diagnosis	ET	PET-MF	PMF	ET	PreMF	PMF	ET	PET-MF	ET	PMF
Treatment at inclusion	Pegylated interferon-alpha	Anagrelide	Pegylated interferon-alpha	Pegylated interferon-alpha	Pegylated interferon-alpha	None	Pegylated interferon-alpha	Pegylated interferon-alpha	Pegylated interferon-alpha	Pegylated interferon-alpha
Splenomegaly at inclusion (Y/N)	N	N	Y	N	Y	Unknown	N	Y	N	Y
*CALR* mutation type	Type 1 (52 bd del)	Type 2(5 bp ins)	Type 1 (52 bd del)	Type 2(5 bp ins)	Type 2(5 bp ins)	Type 2(5 bp ins)	Type 1 (52 bp del)	Type 2(5 bp ins)	Type 1 (52 bp del)	Type 1 (52 bp del)
*CALR*mut VAF at inclusion	33%	43%	44%	18%	41%	41%	23%	40%	1%	49%
Immune response (Y/N)	Y	Y	N	Y	Y	Y	Y	Y	Y	N

### Adverse Events and Safety Profile

All patients experienced AE and these were both local and systemic (**Table 3**). No vaccination related AE ≥ grade 3 were observed. The most common reactions were injection site specific with grade 2 in nine of ten patients at any time. Most common systemic reaction was flu like symptoms in five patients. Two grade 3 AE were registered: One vasovagal reaction during bone marrow biopsy at the end of study, and one patient experienced hypertension before the first vaccine was administered. One autoimmune reaction was registered (see below) but this reaction was not deemed to be triggered by the vaccines.

**Table 3:** Adverse events registered during the trial.

Adverse event	Number of patients	Grade 1	Grade 2	Grade 3
Injection site reactions	10	1	9	0
Flu like symptoms	5	4	1	0
Headache	3	3	0	0
Infection	7	6	1	0
Pruritus	2	2	0	0
Vomiting	1	1	0	0
Mucositis	2	2	0	0
Hot flashes	2	2	0	0
Nausea	2	2	0	0
Myalgia	3	3	0	0
Fever without neutropenia	1	1	0	0
Fatigue	5	2	3	0
Diarrhea	2	2	0	0
Chills	2	1	1	0
Coughing	2	2	0	0
Arthralgia	2	2	0	0
Anorexia	1	0	1	0
ALAT derangement	1	0	1	0
Vertigo	2	1	1	0
Vasovagal reaction	1	0	0	1
Tinnitus	1	1	0	0
Palpitations	1	2	0	0
Pain in extremity	1	1	0	0
Malaise	1	1	0	0
Hypertension	1	0	0	1
Gastrointestinal disorder—other	1	1	0	0
Fall	1	1	0	0
Epistaxis	1	1	0	0
Bruising	1	0	1	0
Allergic rhinitis	2	1	0	0
Atrial fibrillation	1	1	1	0
Acute coronary syndrome	1	1	0	0
Abdominal pain	1	0	1	0

### Clinical Response and Variations in *CALR*mut Variant Allele Frequency

**Figure 1:**
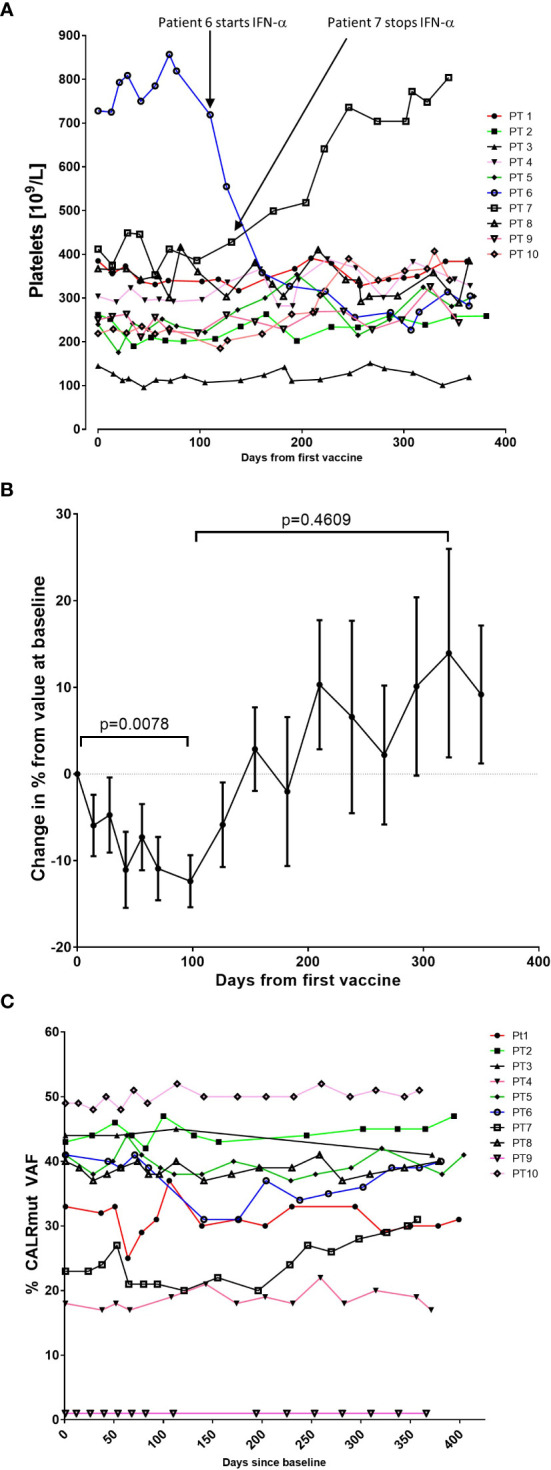
Variation in platelet counts and *CALR*mut variant allele frequency (VAF) during the trial. **(A)** Platelet counts (10^9^/L) for each patient during the trial. **(B)** To get a better impression of the cumulative change in platelet counts, the relative change in platelet counts from baseline was calculated for each patient. Each dot represents the mean change in platelet count in percent relative to the platelet count at baseline. Error bars depict the standard error of the mean. Statistical testing was performed with the Wilcoxon signed-rank test. **(C)** Changes in the *CALR*mut VAF over time.

As the *CALR* mutations mainly affect the megakaryocytic lineage, and patients present with marked thrombocythemia, we expected that the vaccinations would induce a decrease in platelet counts. Looking at each patient individually, we did not demonstrate any decrease in platelet counts (**Figure 1A**). Of note, one patient (patient 6), who did not receive any therapy at inclusion in the trial, was started on IFN-α after 7^th^ vaccination due to high platelet counts. Another patient (patient 7) had to stop IFN-α after 8 vaccinations due to elevated liver enzymes and concomitant development of smooth muscle antibodies (SMA). Before and during the first vaccines, the patient developed increasing levels of alanine aminotransferase (ALAT) below upper normal limit. After 5 vaccinations the level of ALAT reached a maximum of 241 U/l (upper normal limit 70 U/L). SMA were detected in the blood, and examination of archived samples taken at study inclusion prior to vaccine initiation showed low levels of SMA. After IFN-α was stopped the patient continued his vaccinations as planned and normalized his liver function.

We detected a relative decrease in platelet count during the first 100–120 days after the first vaccination that was statistically significant (**Figure 1B**). Platelet levels increased thereafter to a level above baseline levels, but this increase was not statistically significant (**Figure 1B**). We expected to identify a decrease in the *CALR*mut variant allele frequency (VAF) in peripheral blood. The *CALR*mut VAF showed some fluctuations over time, which is also our experience in our normal clinical setting. However, the *CALR*mut VAF displayed no substantial decrease nor increase in any of the patients (**Figure 1C**) even in the patient that displayed a *CALR*mut VAF of only 1% at baseline. As described, patients underwent a full bone marrow examination at baseline and at end of study. No morphological or cytological changes were detected in patients during the vaccination schedule. Analysis of additional mutations quantified by NGS showed that seven of 10 patients harbored additional mutations at baseline and three patients only had a *CALR* mutation with no additional mutations (**Table 4**). However, at end of study one of these three patients had developed an additional mutation. Of note, among the additional mutations identified by NGS, several genes of importance in myeloid cancers such as *ASXL1*, *TET2*, *EZH2*, *SF3B1*, and *DNMT3A* were detected. Three patients displayed a mutation in *TP53*. The majority of patients did not experience marked alterations in mutant VAF as measured by NGS. However, patient 6 showed a decrease in *DNMT3A* (20% VAF to 12% VAF), whereas *ASXL1* increased from 0.66% to 2.1%. Patient 8 was the only patient who demonstrated a marked expansion of a subclone as *GNAS* mutant VAF increased from 0.99% to 10% during the vaccination schedule. Additionally, patient 4 developed a new *STAG2*-mutant subclone and patient 10 developed a *BCOR*-mutant subclone during the vaccination schedule.

**Table 4:** Mutant variant allele frequency (VAF) of *CALR* and additional mutations identified by next generation sequencing.

Patient ID	Mutations at baseline (% VAF)	Mutations at end of study (%VAF)
**1**	CALR (15)	CALR (15)
**2**	CALR (37)	CALR (39)
**3**	CALR (24) TP53 (1)	CALR (26) TP53 (0.5)
**4**	CALR (15)	CALR (14) STAG2 (1.5)
**5**	CALR (32) SF3B1 (0.22)	CALR (32) SF3B1 (1.6)
**6**	CALR (34) DNMT3A (20) EZH2 (3.4) TP53 (0.84) ASXL1 (0.66)	CALR (30) DNMT3A (12) EZH2 (5) TP53 (1.2) ASXL1 (2.1)
**7**	CALR (14) NF1 (1.3)	CALR (17) NF1 (1.2)
**8**	CALR (35) GNAS (0.99)	CALR (35) GNAS (10)
**9**	CALR (1) TP53 (1.02) TET2 (1.4)	CALR (1) TP53(1.4) TET2(1.4)
**10**	CALR (31) SF3B1 (43)	CALR (33) SF3B1 (45) BCOR (1.5)

### Immune Response to the Vaccination Antigen in Peripheral Blood Mononuclear Cells

**Figure 2:**
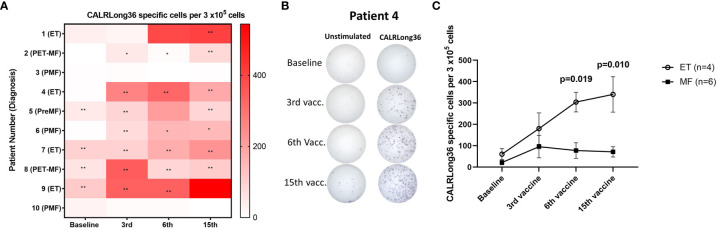
Immune responses to CALRLong36 in IFN-γ ELISPOT. **(A)** Heat map depicting the responses to CALRLong36 in patient peripheral blood mononuclear cells at each time point for all patients. The number of CALRLong36-specific cells was calculated by subtracting the mean spots in the control wells from the mean spots in the peptide-stimulated wells. The analysis was only performed in duplicates for patient 1 and 5 at the 6^th^ vaccination and for patient 9 at the 15^th^ vaccination, which prevented us from performing statistical analysis of the results. All other experiments were performed in triplicates. * Indicates a statistically significant response according to the DFR-rule. ** Indicates a statistically significant response according to the DFR(2x)-rule (38). **(B)** Representative image from **(A, C)** ELISPOT responses over time in patients with essential thrombocythemia (ET) and patients with myelofibrosis (MF) with each dot representing the mean of normalized spots. Error bars depict the standard error of the mean. Statistical testing was performed using the Mann-Whitney test.

Patient PBMC isolated at baseline and after three, six, and 15 vaccinations were analyzed for immune responses against the vaccination antigen using IFN-γ ELISPOT. We detected a DFR2x-defined immune response in four of 10 patients at baseline (**Figure 2A**). Interestingly, the vaccinations induced immune responses in several patients that did not show an immune response at baseline: Seven of 10 patients displayed at least a DFR-defined response after three vaccinations, six of 10 had at least a DFR-defined response after six vaccinations, and seven of 10 had at least a DFR-defined response at end of study (**Figure 2A**). The amplitude of the responses increased over time (**Figure 2B**), but interestingly the response in patients with MF did not increase after the third vaccination, whereas the amplitude of the response in patients with ET increased during the entire vaccination schedule (**Figure 2C**). The difference in response amplitude between patients with ET and PMF were statistically significant at 6^th^ vaccination and at the end of the study (**Figure 2C**). Two patients (patients 3 and 10), both of whom had PMF, did not attain an immune response at all during the vaccination schedule.

**Figure 3:**
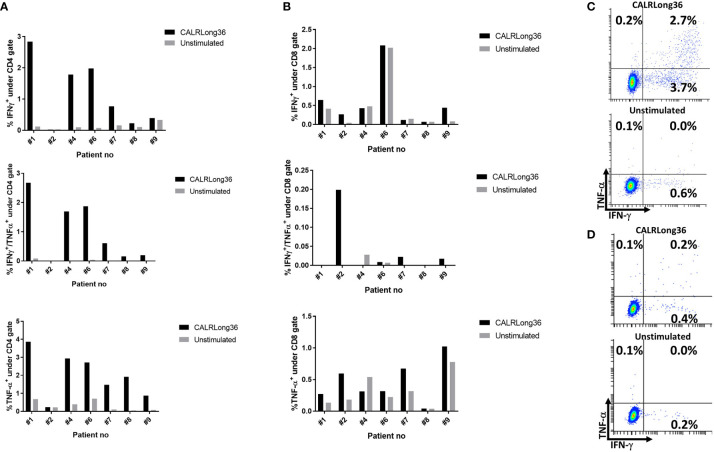
Responses against CALRLong36 identified by intracellular cytokine staining in patients with a response in ELISPOT. **(A)** Responses in CD4^+^-gated T cells with IFN-γ secreting cells (top), IFN-γ/TNF-α secreting cells (middle). and TNF-α secreting cells (bottom). **(B)** Responses in CD8^+^-gated T cells with IFN-γ secreting cells (top), IFN-γ/TNF-α secreting cells (middle), and TNF-α secreting cells (bottom). **(C)** An example of a CD4^+^ T-cell response. **(D)** An example of a CD8^+^ T-cell response.

The phenotype of the cytokine-producing cells was evaluated using intracellular cytokine staining (ICS). From the eight patients who showed an immune response to CALRLong36 in ELISPOT, we analyzed PBMC isolated at the time point where the response in ELISPOT was deemed to be highest. For patients 3 and 10, who did not display an immune response in ELISPOT, we chose to analyze PBMC isolated after 15 vaccinations. Not surprisingly, we did not identify a response in patients who did not show a response in ELISPOT. Additionally, patient 5 had a very low fraction of CD3^+^ T cells, making us unable to gate on a satisfactory number of T cells for our analysis. However, of the remaining seven patients, six displayed a CD4^+^ T-cell response to stimulation with CALRLong36 (**Figures 3A, C**), and two patients displayed a CD8^+^ T-cell response (**Figures 3B, D**). Patient 7 demonstrated both a CD4^+^ and a CD8^+^ T-cell response, whereas patient 2 demonstrated only a CD8^+^ T-cell response. Patient 2 provided informed consent for us to isolate PBMC at several time points after the end of study and showed a sustained CD8^+^ T-cell response to CALRLong36 even at 37 and 49 weeks after the last vaccination (**Supplementary Figure 5**).

### Delayed-Type Hypersensitivity and Response in Skin-Infiltrating Lymphocytes

**Figure 4:**
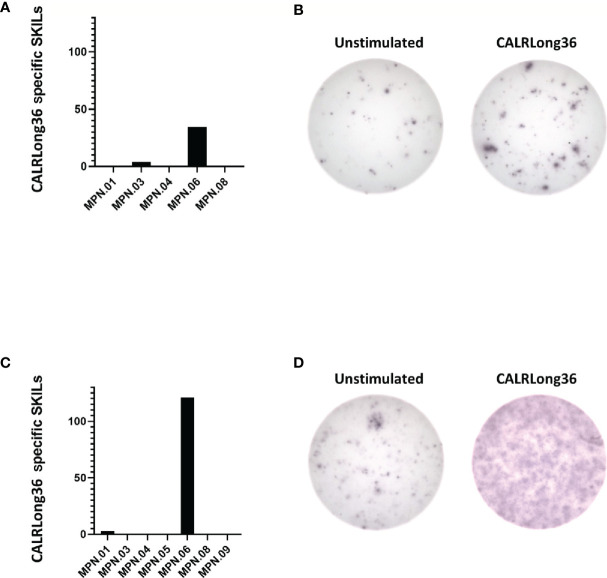
Responses in skin-infiltrating lymphocytes (SKILs) against CALRLong36. SKILs were harvested as described in the Materials and Methods section and were then expanded in either high-dose IL-2 (6,000 U/mL) culture medium or low-dose IL-2 (100 U/mL) culture medium. Cells were harvested and analyzed by IFN-γ ELISPOT for a response against CALRLong36. **(A)** Normalized numbers of cells specific to CALRLong36 in SKILs from patient 1, 3, 4, 6 and 8 cultured in low dose IL-2. **(B)** Response in SKILs from patients 6 expanded in low dose IL-2. **(C)** Normalized numbers of cells specific to CALRLong36 in SKILs from patient 1, 3, 4, 5, 6, 8 and 9 cultured in high dose IL-2. **(D)** Response in SKILs from patients 6 expanded in high dose IL-2.

Ten patients received intradermal injections with CALRLong36 as described in ***Materials and Methods***. Of note, none of the patients demonstrated an induration at the site of intradermal injections. In order to investigate if the injections could have induced a migration of CALRLong36-specific T cells to the injection site, 10 patients were subjected to a punch biopsy at the injection site. Punch biopsies were cultured as described previously (39) to expand specific T cells in the biopsy. Surprisingly, even though none of the patients displayed an induration at the injection sites, we were able to expand skin-infiltrating lymphocytes (SKILs) from five patients by culturing with low dose IL-2 (100 U/mL) and SKILs from seven patients by culturing with high dose IL-2 (6,000 U/mL). The SKILs were tested in IFN-γ ELISPOT for response to CALRLong36. Only patient 6 displayed a response (**Figure 4**); the responses in the remaining patients were either absent or the background was too high (**Supplementary Figure 6**).

### Phenotype of Peripheral Blood Mononuclear Cells During the Vaccination Schedule

**Figure 5:**
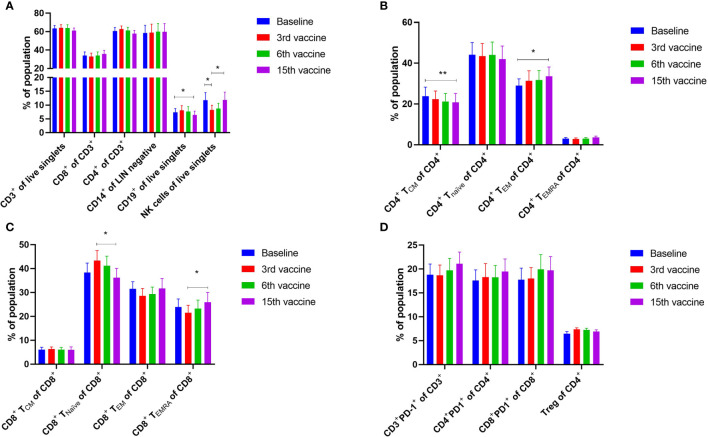
Phenotyping of peripheral blood mononuclear cells (PBMC) and T cells by fluorescence-activated cell sorting. **(A)** Quantification of the mean PBMC subsets in peripheral blood of vaccinated patients. **(B)** Quantification of CD4^+^ central memory T cells (T_CM_), CD4^+^ naïve T cells (T_naïve_), CD4^+^ effector memory T cells (T_EM_), and CD4^+^ effector memory T cells re-expressing CD45RA (T_EMRA_). **(C)** Quantification of CD8^+^ central memory T cells (T_CM_), CD8^+^ naïve T cells (T_naïve_), CD8^+^ effector memory T cells (T_EM_), and CD8^+^ effector memory T cells re-expressing CD45RA (T_EMRA_). **(D)** Expression levels of PD-1 on T cells and CD4^+^ and CD8^+^ T cells, and number of regulatory T cells (Treg). Statistical testing was performed using the Wilcoxon signed-rank test. Bars represent standard error of the mean. * denotes p ≤ 0.05, ** denotes p ≤ 0.01.

**Figure 6:**
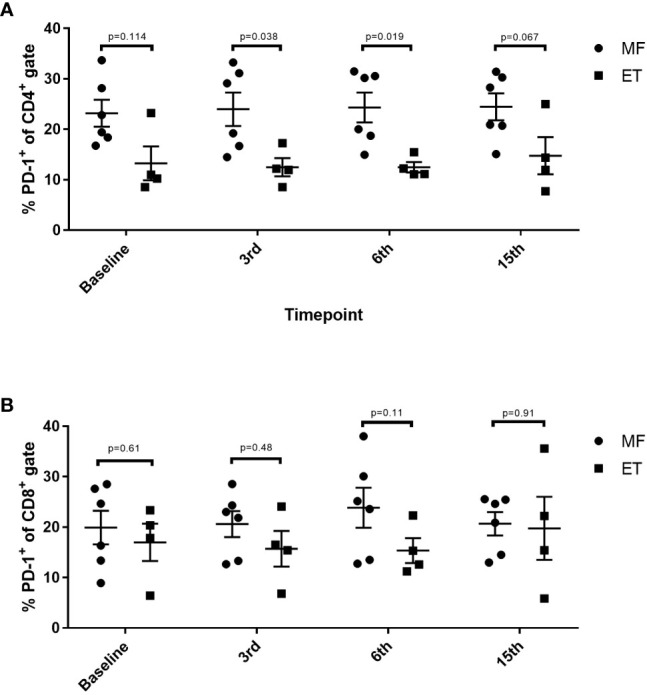
Changes in levels of PD-1 expression in CD4^+^ and CD8^+^ T cells in patients with essential thrombocythemia (ET) and primary myelofibrosis (PMF) during the vaccination schedule. **(A)** PD-1 expression on CD4^+^ T cells. **(B)** PD-1 expression on CD8^+^ T cells. Statistical testing was performed using the Mann-Whitney test. Bars represent standard error of the mean.

**Figure 7:**
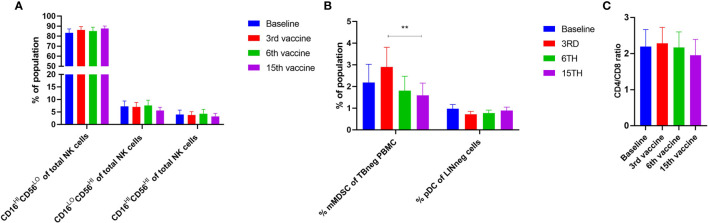
Phenotyping of NK cells and other cells fractions in PBMC by fluorescence-activated cell sorting. **(A)** Levels of different NK cell subsets. **(B)** Alterations in the levels of monocytic MDSC (mMDSC) and plasmacytoid dendritic cells (pDC). **(C)** Quantification of the CD4^+^/CD8^+^ ratio during the trial. Statistical testing was performed using the Wilcoxon signed-rank test. Bars represent standard error of the mean. ** denotes p ≤ 0.01.

Alterations in the composition of immune cells in peripheral blood were analyzed during the vaccination schedule. No alterations were identified in the number of T cells in general, nor in the numbers of CD4^+^ and CD8^+^ T cells. Neither did we detect any changes in the levels of CD14^+^ monocytes. B cells increased slightly during the study, but returned to baseline levels at the end of the trial. NK cells decreased initially, but towards the end of the study they reached their level at baseline (**Figure 5A**). Interestingly, we identified a statistically significant decrease in the number of circulating CD4^+^ central memory T cells (T_CM_) in combination with a statistically significant increase in the number of CD4^+^ effector memory T cells (T_EM_) (**Figure 5B**). Changes in the CD8^+^ T-cell fraction were not dramatic, but we detected a somewhat paradoxical increase in the number of CD8^+^ naïve T cells (T_naïve_) during the vaccination schedule, and levels of T_naïve_ only returned to baseline levels at the end of the study period (**Figure 5C**). Conversely, the number of CD8^+^ T_EMRA_ decreased after the first three vaccinations, after which it increased to reach the levels at baseline (**Figure 5C**). Levels of PD-1 on both T cells in total and in CD4^+^ and CD8^+^ T cells remained constant, as did the levels of regulatory T cells (Treg) (**Figure 5D**). As PD-1 expression is increased on T cells in patients with MPN (40, 41) we sought to analyze differences in PD-1 expression between ET- and PMF-patients. Most interestingly, we showed that at the third and sixth vaccination, CD4^+^ T cells from patients with MF expressed significantly higher levels of PD-1 compared with patients with ET (**Figure 6A**). A difference in expression levels was also detected at baseline and at the 15^th^ vaccination, but the difference was not statistically significant (**Figure 6A**). On the other hand, expression of PD-1 by CD8^+^ T cells did not differ between patients with ET and PMF (**Figure 6B**). No significant changes were identified in the different NK cell subsets (**Figure 7A**). Of note, levels of monocytic myeloid-derived suppressor cells (mMDSC) increased initially but decreased to a level lower than that at baseline (**Figure 7B**), and the CD4^+^/CD8^+^ T-cell ratio remained constant throughout the study (**Figure 7C**).

## Discussion

In order to attain a clinical response using cancer immune therapy, it is of utmost importance that the tumor cells express immunogenic antigens that may be targeted by specific T cells (42). The *RAS* mutations generate several immunogenic neo-antigens that are targets of specific T cells, and therapeutic cancer vaccines targeting these neo-antigens have shown a survival benefit in patients with an immune response to the vaccine epitope (33, 43). Vaccination of AML patients with immunogenic tumor-associated antigens such as Wilms tumor antigen-1 (WT1) and NY-ESO have also demonstrated a survival benefit for patients with an immune response (44, 45). The *NPM1* mutations identified in a substantial proportion of patients with AML generate highly immunogenic antigens (46), and it has been shown that patients with an intact immune response to NPM1-derived neo-antigens have significantly longer overall survival compared with patients without a response (47). As such, the existence of a neo-antigen-specific immune response is believed to be highly important in attaining a therapeutic effect. In recent years, research has mainly focused on the potentially large number of patient-specific, mutated neoantigens. They comprise the predicted products of the numerous mutations revealed by exome sequencing of primary tumors, and it was demonstrated that it is not only the number of mutations that predict outcome but also the immunogenicity of the antigens generated by the somatic mutations (48).

We have shown earlier that patients with *CALR*mut MPN harbor T cells specific to neo-antigens derived from the mutant CALR C-terminus. However, patient T-cell responses are less frequent and weaker than the responses identified in healthy donors. This could indicate that patients with *CALR*mut MPN harbor an exhausted *CALR*mut-specific immune response, and we speculated that therapeutic cancer vaccination with a *CALR*mut-derived peptide could enhance the tumor-specific immune response in patients and lead to a clinical response. As the *CALR* mutations have been shown to be early mutations residing in the highly undifferentiated HSC (21), a strong *CALR*mut-specific immune response in patients may be of concern, as a strong immune response could potentially eradicate all *CALR*mut HSC in the patients, and thus, result in bone marrow failure. Only a few side effects were identified in our study, with only two grade 3 AE. One patient developed hypertension during the trial, but this occurred before the administration of the first vaccine, thus it is impossible that the vaccines elicited this AE. Another patient experienced a vasovagal episode just prior to the end-of-study bone marrow biopsy. The episode could be explained simply by the fact that bone marrow biopsies are highly uncomfortable and somewhat painful for the patients. Apart from these, no severe AE (SAE) were observed. The most frequent AE registered in this trial, infection, fatigue and myalgia, are complaints that are common in patients with MPN. The injection-site reactions experienced by all patients and the flu-like symptoms experienced by 50% of the patients are likely reactions to the montanide adjuvant in the vaccine. The occurrence of these AE could actually be regarded as a beneficial sign, as they indicate that a proper inflammatory response to the vaccine was induced. In conclusion, this vaccination trial adds weight to the notion that cancer vaccines only very rarely induce SAE (49). The patient that experienced a transient increase in ALAT showed evidence of increased SMA even before study entry, thus this AE is not believed to be induced by the vaccines.

Given the immunogenicity of the *CALR* mutations, we expected the vaccines to induce a clinical response in patients. As the *CALR* mutations mainly affect the megakaryocytic lineage (50–53) and patients with *CALR*mut MPN display higher platelet counts than their *JAK2*V617F^+^ counterparts (54, 55), we expected that a clinical response would be identified by a decline in platelet counts. Indeed, we detected a decline in platelet counts during the first 100 days, after which the numbers increased. Importantly, apart from patient 6 and 7, no patients had any adjustments in their MPN-directed therapy during the trial. Thus, this cannot explain the changes detected in platelet counts. A potential cause of the decline in platelets is a tumor-specific immune response that targets the megakaryocytes and thus reduces platelet production. Importantly, the subsequent increase in platelet counts coincided with the conversion of biweekly vaccinations into monthly vaccinations. To our belief, the decline in platelet counts during the first period of time could be explained by a tumor specific T-cell response and certainly needs further investigation in future trials.

The decline in platelet counts did not translate into a decline in the *CALR*mut VAF. In daily practice the VAF oscillates over time but largely remains constant, which was also recorded in our cohort. Theoretically, some patients were more likely to display a response to the vaccines than others. Thus, patient number 6 was not receiving any treatment at inclusion, but after seven vaccinations it was decided to start treatment with IFN-α (45 µg weekly) due to a high platelet count. As IFN-α is a highly immunostimulatory cytokine, we believed that the addition of IFN-α to the vaccines would induce a molecular response. Patient number 9 displayed a very low *CALR*mut VAF of only 1%. Earlier reports on therapeutic cancer vaccines suggest that patients with a low tumor burden are more likely to benefit from therapy (56, 57). Since patient 9 had a relatively low tumor burden we believed that the low number of tumor cells would not impede the tumor-specific immune response. Despite the low tumor burden in this patient, the vaccines did not induce a clinical nor molecular response. However, it is intriguing to consider, whether the lack of responses might be attributed to the additional mutations in both *TP53* and *TET2*, rendering the *CALR*-mutant clone resistant to immune mediated clearing.

Our results clearly demonstrate that a lack of clinico-hematological and molecular response to the CALRmut-vaccine is not explicitly due to incompetent CALRLong36-specific immune responses. Thus, eight patients displayed strong immune responses to CALRLong36. Of these eight patients, four showed an immune response at baseline that was later enhanced by the vaccines. In the remaining four patients who displayed an immune response to CALRLong36, the response was absent at baseline but induced during the course of the trial. Two patients, both of whom have PMF, did not display an immune response against the vaccine. Interestingly, we found that the immune response in patients with ET increased during the vaccination course, in contrast to patients with PMF in whom the amplitude of the immune response remained stable after the first three vaccinations. We believe that this could be due to the more severe immune dysfunction in patients with PMF, and the results are on par with our earlier results showing that patients with ET have more frequent responses than patients with PMF (23, 58, 59). Recently it was demonstrated that monocyte-derived dendritic cells (moDC) from patients with PMF display lower levels of costimulatory molecules compared with healthy-donor moDC, and that these moDC have inferior priming potential (60). This could explain the weaker responses identified in patients with PMF. In contrast, T cells from patients with *CALR*mut MPN are more prone to activation, and Treg from *CALR*mut patients display a lower inhibitory potential than Treg from *CALR*wt patients (60), which should counteract the deficiency in moDC. We hypothesized that the differences in immune responses might be partially explained by the occurrence of additional mutations, however we did not identify any association between lack of immune response and additional mutations identified by NGS.

Earlier reports have shown that the majority of *CALR*mut-specific immune responses identified are CD4^+^ T-cell responses (22, 23, 41, 61). In this trial, six of seven patients showed a CD4^+^ T-cell response, and one of these displayed a CD8^+^ T-cell response as well. One patient displayed only a CD8^+^ T-cell response, and this response was sustained even after cessation of vaccination. Earlier trials have shown that a sustained tumor-specific CD8^+^ T-cell response is important in attaining a clinical response (62), and the apparent lack of CD8^+^ T-cell responses could explain the lack of clinical results. However, the low frequency of CD8^+^ T-cell responses could also be due to our experimental setup during the ICS, as the cells are only allowed to incubate with the peptide for 5 h, thus leaving only a little time for antigen-presenting cells to process and present possible CD8^+^ epitopes. Arshad et al. showed that the *CALR* mutations inhibit the presentation of peptides by human leukocyte antigen (HLA)-I molecules (63), as the CALR protein is an important chaperone in the assembly of the peptide:HLA-I complex. The low number of CD8^+^ T-cell responses identified could thus also be explained by the inability of patient antigen-presenting cells to process and present high-quality HLA-I-restricted epitopes. We do therefore find it noteworthy that two of the seven patients analyzed showed a CALRmut-specific CD8^+^ T-cell response.

Earlier peptide vaccination trials have evaluated the immune response not only by measuring response in PBMC but also by measuring the immune response in SKILs and the occurrence of a DTH induration from the vaccination antigen after intradermal injection. Collectively, patients with tumor-specific T cells at the DTH site have a greater chance of a clinical response to vaccination (64, 65). Strikingly, no patients displayed a DTH induration in this trial. We performed punch biopsy at the site of the intradermal injection in all patients and were able to grow SKILs from eight patients. These SKILs were tested in IFN-γ ELISPOT assays and only one patient displayed a response to CALRLong36. The apparent lack of DTH in our patient cohort is striking. First, because the antigen is highly immunogenic, and second because the majority of patients receiving intradermal injections with antigen in other trials show a DTH. As no patients demonstrated a DTH, we speculate that this was due to a general feature, either of CALRLong36 that prevents it from being processed and presented by dermal dendritic cells, or of MPN patients in general, who might harbor a deficiency in the dermal immune system that renders the dermal immune cells hyporesponsive to stimuli. Our current trial testing vaccinations with epitopes derived from programmed death ligand (PD-L)-1 and arginase (ARG)-1 (NCT04051307) will shed further light on this question. Of note, patients with psoriasis, an autoimmune skin disease primarily driven by T cells (66), are at an elevated risk of developing PMF (67), which could indicate that the dermal immune system in at least patients with PMF is dysregulated.

As therapeutic cancer vaccines generally do not induce any systemic effects, a marked alteration in the immune phenotype of patients was not expected. However, as patients with mutated *CALR* are heterozygous for the mutations, and the mutant VAF reaches almost 50% in all patients, essentially all cells of myeloid origin are believed to carry the mutation. Additionally, we have shown that T and B cells may also harbor the mutation (68). As such, T-cell mediated targeting of the *CALR* mutations has the potential to have a noticeable effect on the phenotype of circulating immune cells. Alterations in the phenotype of effector or suppressor immune cells could also be expected. We identified an increase in the number of CD4^+^ T_EM_, whereas CD4^+^ T_CM_ levels declined. We believe that these data are highly interesting, as they could imply that the immune system reaches a more effector-like phenotype as T_CM_ differentiate into T_EM_, which are more active in cytotoxic killing of tumor cells (69, 70). CD8^+^ T_naïve_ increased initially, and then declined. The decline might be explained by the priming of T_naïve_ by the vaccine, while the subsequent increase in CD8^+^ T_EMRA_ might be explained by the sustained presentation of antigen to T cells that subsequently terminally differentiate and become hyporesponsive. Riley et al. monitored the phenotype of circulating immune cells in patients with *JAK2*V617F^+^ PV treated with IFN-α extensively and identified an increase in Treg numbers in these patients (71). Another study demonstrated an expansion of CD56^hi^ NK cells in patients receiving IFN-α for more than a year (72). We did not identify any changes in the levels of Tregs nor in the numbers of NK cell subsets, but the total number of NK cells declined after the first vaccines, after which the levels increased to baseline levels after 15 vaccinations. As noted above, the main *CALR*mut cells in the PBMC fraction are monocytes, and a clinical response to therapy would likely be reflected by decreasing numbers of CD14^+^ monocytes in the PBMC fraction, but such changes were not identified. Monocytic myeloid-derived suppressor cells (mMDSC) are cells of myeloid origin that are highly immunosuppressive (73). Patients with MPN have elevated levels of mMDSC in their peripheral blood (74), which could impede the tumor-specific immune response. Patients experienced a small increase in peripheral blood mMDSC followed by a decrease, a phenomenon also identified in CD19^+^ B cells. Neither of these were highly significant and to our thinking are not alterations reflecting the effect of the vaccinations. Earlier studies of the expression of PD-1 on T cell in patients with MPN have shown that PD-1 expression is increased on patient T cells compared to healthy donor T cells (40, 41). We did not have a healthy donor cohort for comparison in this study, but compared our subjects’ levels with the normal levels identified in earlier reports (40, 41). Looking at PBMC from *CALR*mut patients only, Cimen-Bozkus et al. found that 19.2% of CD3^+^ T cells were PD-1^+^, which is comparable to our results (41). The PD-1 expression on CD4^+^ and CD8^+^ T-cells are also comparable to our results, and in another report analyzing PBMC from both *JAK2*V617F^+^ and *CALR*mut patients, the numbers of PD-1^+^ CD4^+^ and CD8^+^ T-cells were even higher (40). Of note, both reports show that patient T cells have significantly higher expression of PD-1 compared to healthy-donor T cells, and several additional exhaustion markers such as CTLA-4 have also been shown to be enhanced in T cells from *CALR*mut patients (41).

As the majority of responses to mutant CALR in both patients and healthy donors are CD4^+^ T cell responses (23, 41, 61), and mutant CALR is expressed on the surface of mutant cells (53), one would expect the occurrence of CALRmut specific antibodies. However, the occurrence of such antibodies has not been reported. Of note, *CALR*mut cells secrete mutant CALR to the extracellular compartment (75, 76), and patients with PMF exhibit elevated levels of circulating CALR compared to healthy donors (77). This could explain the apparent lack of antibodies specific to mutant CALR, as circulating mutant CALR could neutralize the formed antibodies making these undetectable and additionally impede on the anti-tumor effect of the antibodies. Other possible effects of circulating mutant CALR could be that the mutant protein induces tolerance in specific T cells due to chronic antigen stimulation.

One of the main conclusions from this trial is that even though therapeutic cancer vaccination against an antigen derived from the *CALR* exon 9 mutations induces T-cell responses specific to the vaccination antigen, this does not translate into a molecular response. Thus, future trials employing therapeutic cancer vaccinations against mutant *CALR* should aim to combine the vaccines with other treatments. This should be highly feasible as the vaccination is well tolerated. One obvious treatment to combine with a vaccine would be IFN-α. IFN-α enhances the Th1 immune response (8) and, specifically in MPN, the expression of genes related to antigen processing and presentation (78). However, the majority of patients in our trial were receiving IFN-α, and we cannot rule out that the dose administered is too low to enhance the effect of the vaccine. Immune-checkpoint inhibitors (ICPI) have demonstrated strong clinical potential in several solid cancers and in the treatment of Hodgkin lymphoma (79), and the potential of using ICPI to treat MPN is highly intriguing. A recent report showed that treatment of *CALR*mut MPN patients with ICPI induces an immune response in patient PBMC stimulated *in vitro* with both the peptide and ICPI (41), adding impetus to the notion that ICPI can be used to enhance a neo-antigen-specific immune response in patients with *CALR*mut MPN. However, data on the clinical effect of ICPI are yet to be reported. Considering the immense number of adverse events observed following treatment with ICPI, caution should be used when treating patients with non-advanced MPN, as several less toxic treatment modalities are at hand for these patients. It has been shown that patients with *CALR*mut PMF have better overall survival after alloHSCT compared with patients with *CALR*wt PMF (80). This could be explained by recognition and clearing of residual *CALR*mut cells by donor T cells. In the setting of alloHSCT, donor lymphocyte infusion (DLI) can be used for patients who do not attain complete remission after alloHSCT. DLI acts by enhancing the graft-versus-leukemia effect and has shown its potency in the treatment of PMF (81). Interestingly, a patient with *NPM1*-mutant AML was brought into remission by DLI, and after engraftment the authors detected NPM1mut-specific T-cell responses by ELISPOT (82). One probable mechanism beyond the therapeutic effect of the DLI may be that NPM1mut-specific T cells in the infusion product have recognized and killed residual AML blasts. Vaccination of HSCT donors before harvest of the DLI product could potentially enhance the number of CALRmut-specific T cells in the infusion product, thus increasing the likelihood of DLI-mediated clearing of residual *CALR*mut cells. Another highly active compound in MPN is ruxolitinib which is a very potent anti-inflammatory drug that inhibits JAK1-2 signaling. Ruxolitinib decreases constitutional symptoms and reduces splenomegaly in a majority of patients (83). However, combination of ruxolitinib and vaccines is not believed to induce clinico-hematological responses in patients since ruxolitinib, due to its JAK1-2 inhibiting properties, attenuates the functionality of dendritic cells, NK cells and T cells (84–86). Hence, the combination of ruxolitinib with cancer immune therapeutic modalities is not believed to result in clinical responses.

As noted above, several studies have shown that patients with MPN harbor an increased number of MDSC in peripheral blood, and patient T cells express elevated amounts of PD-1 in conjunction with elevated amounts of PD-L1 (40). Together, these factors render the immune environment in MPN highly immunosuppressive, which could also explain the lack of response to the vaccines. As mentioned, one way to circumvent this immune suppression would be ICPI. However, it has recently been reported that the immune system itself harbors autoreactive anti-regulatory T cells that are able to kill regulatory immune cells through the recognition of regulatory proteins and enzymes (87). T cells specific to important immunoregulatory molecules such as PD-L1, PD-L2, indoleamine 2,3-dioxygenase (IDO), and ARG-1/2 have been described (88–92), and vaccination with IDO- and PD-L1-derived epitopes have already shown clinical benefit to patients with stage IV non-small-cell lung cancer and metastatic melanoma (93, 94). As patients with MPN have increased levels of several of these regulatory proteins, we believe that induction of an anti-regulatory immune response through vaccination with one or several anti-regulatory T cell epitopes will enhance the anti-regulatory immune responses which ultimately will lower the immune suppression in patients (95). In combination with *CALR*mut-specific therapeutic cancer vaccination, these anti-regulatory vaccines have the potential to induce a clinical response, especially as patients with MPN harbor T cells specific to both PD-L1- and ARG-1-derived epitopes (58, 59). This finding spurred us to initiate a phase I/II vaccination trial testing the combination of PD-L1- and ARG-1-derived epitopes in patients with ET and PV (NCT04051307).

Immune tolerance might be another barrier to the vaccines having an effect. Patients with MPN may live for decades with their disease. As chronic antigen stimulation has been shown to induce tolerance in antigen-specific T cells, we speculate that specific T cells in patients with *CALR*mut MPN are exhausted and the effect of vaccines is simply not enough to revert this tolerance. Recently, a Danish population-based study showed that healthy donors may harbor *CALR* exon 9 mutations with a low VAF without showing any signs of MPN (96). Thus, we believe that *CALR*mut MPN may evolve due to immune escape after prolonged exposure of CALRmut antigens to T cells, and speculate that therapeutic cancer vaccination at a very early stage, if possible the preclinical stage, will induce a clinical effect. Of note, *CALR*mut healthy donors identified in the study by Cordua et al. harbor CALRmut-specific immune responses, supporting the notion that these individuals hold the *CALR*mut HSC at bay through T-cell-mediated elimination of mutant cells (97). Thus, we suggest early up-front vaccination therapy in order to minimize the risk of T-cell exhaustion and concurrently induce a T-cell response when the tumor burden is still relatively low. Although therapeutic cancer vaccination in healthy individuals is controversial, it is a feasible option given the low frequency of serious AE identified in this trial.

In conclusion, this clinical phase I vaccination trial with an epitope derived from the *CALR* exon 9 mutations showed that the vaccine is safe and tolerable. The vaccines either induced or enhanced an existing CALRmut-specific immune response in the majority of patients. However, the immune responses did not translate into a clinical response as no patients experienced any improvement in their disease status. Since the vaccines have a good safety profile, we suggest combining the vaccine with other immune therapeutic modalities in order to induce a clinical response.

## Data Availability Statement

The data sets presented in this article are not readily available because Danish Law and the General Data Protection Rules prohibits this. Requests to access the data sets should be directed to molecular biologist VS at vihs@regionsjaelland.dk.

## Ethics Statement

The studies involving human participants were reviewed and approved by Zealand Region Ethics Committee. The patients/participants provided their written informed consent to participate in this study.

## Author Contributions

JHG conducted the trial, analyzed the data, and wrote the manuscript. MOH concieved the project, performed experiments, analyzed data, and wrote the manuscript. NJ analyzed data. UK analyzed data. SW-B performed experiments and analyzed the data. CS and MC recruited, treated patients, and analyzed data. LG and MB performed experiments and analyzed data. JK analyzed data. MH analyzed data and provided vital reagent. SK and NC analyzed data. GN, JP, LK, and VS performed experiments, analyzed data, and wrote the manuscript. ÖM analyzed data and provided vital reagents. IS analyzed data and provided vital reagent. HH and MHA conceived the project, analyzed data, and wrote the manuscript. All authors contributed to the article and approved the submitted version.

## Funding

This study was supported in part by Kræftens Bekæmpelse grant number R149-A10159-16-S47 and a pre-seed grant from the Novo Nordisk Foundation.

## Conflict of Interest

MOH, HH, and MA have filed a patent regarding the *CALR* exon 9 mutations as a target for cancer immune therapy. The patent has been transferred to University Hospital Zealand, Zealand Region and Copenhagen University Hospital at Herlev, Capital Region according to Danish Law concerning inventions made at public research institutions.

The remaining authors declare that the research was conducted in the absence of any commercial or financial relationships that could be construed as a potential conflict of interest.

## Supplementary Material

Supplementary Figure 1 Detailed in- and exclusion criteria.

Supplementary Figure 2 Gant chart showing the vaccination schedule and additional examination/analyses. Abbreviations: V=vaccine, HB=health blood sample, SB=study blood sample, PE=physical examination, ECG=electrocardiogram, BM=bone marrow examination, DTH=delayed type hypersensitivity, NGS=next generation sequencing

Supplementary Figure 3 Gating strategy for identification of immune cell subsets in vaccinated patients.

Supplementary Figure 4 List of antibodies used for identification of immune cell substest in vaccinated patients.

Supplementary Figure 5 Sustained CD8^+^ T-cell response in patient 2 even after cessation of vaccination, identified by intracellular cytokine staining. **(A)** CD8^+^ T-cell response in patient PBMC at 37 weeks after cessation of therapy. **(B)** CD8^+^ T-cell response in patient PBMC at 49 weeks after cessation of therapy.

Supplementary Figure 6 Response in skin-infiltrating lymphocytes showing response in both the CALRLong36-stimulated wells and the negative control wells.

## References

[1] Campbell PPJ Green AAR. The Myeloproliferative Disorders. N Engl J Med (2006) 355:2452–66. 10.1056/NEJMra063728 17151367

[2] Spivak JL. Myeloproliferative Neoplasms. N Engl J Med (2017) 376:2168–81. 10.1056/NEJMra1406186 28564565

[3] Arber DA Orazi A Hasserjian R Borowitz MJ Le Beau MM Bloomfield CD. The 2016 revision to the World Health Organization classification of myeloid neoplasms and acute leukemia. Blood (2016) 127:2391–406. 10.1182/blood-2016-03-643544.The 27069254

[4] Wolanskyj AP Schwager SM Mcclure RF Larson DR Tefferi A. Essential Thrombocythemia Beyond the First Decade : Life Expectancy, Long-term Complication Rates, and Prognostic Factors. Mayo Clin Proc (2006) 81:159–66. 10.1016/S0025-6196(11)61664-9 16471068

[5] Mesa RA Silverstein MN Jacobsen SJ Wollan PC Tefferi A. Population-based incidence and survival figures in essential thrombocythemia and agnogenic myeloid metaplasia: An olmsted county study, 1976-1995. Am J Hematol (1999) 61:10–5. 10.1002/(SICI)1096-8652(199905)61:1<10::AID-AJH3>3.0.CO;2-I 10331505

[6] Deeg HJ Gooley TA Flowers MED Sale GE Slattery JT Anasetti C. Allogeneic hematopoietic stem cell transplantation for myelofibrosis. Transplantation (2003) 102:3912–8. 10.1182/blood-2003-06-1856.Supported 12920019

[7] Barbui T Barosi G Birgegard G Cervantes F Finazzi G Griesshammer M. Philadelphia-negative classical myeloproliferative neoplasms: critical concepts and management recommendations from European leukemiaNet. J Clin Oncol (2011) 29:761–70. 10.1200/JCO.2010.31.8436 PMC4979120 21205761

[8] Belardelli F Ferrantini M Proietti E Kirkwood JM. Interferon-alpha in tumor immunity and immunotherapy. Cytokine Growth Factor Rev (2002) 13:119–34. 10.1016/s1359-6101(01)00022-3 11900988

[9] Talpaz M Hehlmann R Quintás-Cardama A Mercer J Cortes J. Re-emergence of interferon-α in the treatment of chronic myeloid leukemia. Leukemia (2013) 27:803–12. 10.1038/leu.2012.313 PMC3703612 23238589

[10] Kiladjian J Cassinat B Chevret S Turlure P Cambier N Roussel M. Pegylated interferon-alfa-2a induces complete hematologic and molecular responses with low toxicity in polycythemia vera. Blood (2008) 112:3065–72. 10.1182/blood-2008-03-143537 18650451

[11] Rank CU Bjerrum OW Larsen TS Kjær L de Stricker K Riley CH. Minimal residual disease after long-term interferon-alpha2 treatment: a report on hematological, molecular and histomorphological response patterns in 10 patients with essential thrombocythemia and polycythemia vera. Leuk Lymphoma (2016) 57:348–54. 10.3109/10428194.2015.1049171 25956046

[12] Hasselbalch HC Holmström MO. Perspectives on interferon-alpha in the treatment of polycythemia vera and related myeloproliferative neoplasms: minimal residual disease and cure? Semin Immunopathol (2019) 41:5–19. 10.1007/s00281-018-0700-2 30203226 PMC6323070

[13] Kiladjian J-J Giraudier S Cassinat B. Interferon-alpha for the therapy of myeloproliferative neoplasms: Targeting the malignant clone. Leukemia (2015) 30:1–6. 10.1038/leu.2015.326 26601783

[14] Barosi G. An Immune Dysregulation in MPN. Curr Hematol Malig Rep (2014) 9:331–9. 10.1007/s11899-014-0227-0 25139710

[15] Holmström MO Hasselbalch HC Andersen MH. Cancer immune therapy for Philadelphia chromosome-negative chronic myeloproliferative neoplasms. Cancers (Basel) (2020) 12:1–21. 10.3390/cancers12071763 PMC7407874 32630667

[16] Villarino AV Kanno Y Ferdinand JR O’Shea JJ. Mechanisms of Jak/STAT Signaling in Immunity and Disease. J Immunol (2015) 194:21–7. 10.4049/jimmunol.1401867 PMC4524500 25527793

[17] Levine RL Wadleigh M Cools J Ebert BL Wernig G Huntly BJP. Activating mutation in the tyrosine kinase JAK2 in polycythemia vera, essential thrombocythemia, and myeloid metaplasia with myelofibrosis. Cancer Cell (2005) 7:387–97. 10.1016/j.ccr.2005.03.023 15837627

[18] Kralovics R Passamonti F Buser AAS Teo S-SS-S Tiedt R Passweg JRJ. A Gain-of-Function Mutation of JAK2 in Myeloproliferative Disorders. N Engl J Med (2005) 352:1779–90. 10.1056/NEJMoa051113 15858187

[19] Pardanani AD Levine RL Lasho T Pikman Y Mesa RA Wadleigh M. MPL515 mutations in myeloproliferative and other myeloid disorders: A study of 1182 patients. Blood (2006) 108:3472–6. 10.1182/blood-2006-04-018879 16868251

[20] Klampfl T Gisslinger H Harutyunyan AS Nivarthi H Rumi E Milosevic JD. Somatic mutations of calreticulin in myeloproliferative neoplasms. N Engl J Med (2013) 369:2379–90. 10.1056/NEJMoa1311347 24325356

[21] Nangalia J Massie CE Baxter EJ Nice FL Gundem G Wedge DC. Somatic CALR mutations in myeloproliferative neoplasms with nonmutated JAK2. N Engl J Med (2013) 369:2391–405. 10.1056/NEJMoa1312542 PMC3966280 24325359

[22] Holmstrom MO Martinenaite E Ahmad SM Met O Friese C Kjaer L. The calreticulin (CALR) exon 9 mutations are promising targets for cancer immune therapy. Leukemia (2018) 32:429–37. 10.1038/leu.2017.214 28676668

[23] Holmström MO Riley CH Svane IM Hasselbalch HC Andersen MH Holmstrom MO. The CALR exon 9 mutations are shared neoantigens in patients with CALR mutant chronic myeloproliferative neoplasms. Leukemia (2016) 30:2413–6. 10.1038/leu.2016.233 27560107

[24] Coulie PG Van den Eynde BJ van der Bruggen P Boon T. Tumour antigens recognized by T lymphocytes: at the core of cancer immunotherapy. Nat Rev Cancer (2014) 14:135–46. 10.1038/nrc3670 24457417

[25] Melief CJM van Hall T Arens R Ossendorp F van der Burg SH. Therapeutic cancer vaccines. J Clin Invest (2015) 125:1–12. 10.1172/JCI80009 26214521 PMC4588240

[26] Prior IA Lewis PD Mattos C. A comprehensive survey of ras mutations in cancer. Cancer Res (2012) 72:2457–67. 10.1158/0008-5472.CAN-11-2612 PMC3354961 22589270

[27] Qin H Chen W Takahashi M Disis ML Byrd DR McCahill L. CD4+ T-Cell Immunity to Mutated ras Protein in Pancreatic and Colon Cancer Patients. Cancer Res (1995) 55:2984–7. 7606715

[28] Gedde-Dahl T Eriksen JA Thorsby E Gaudernack G. T-cell responses against products of oncogenes: Generation and characterization of human T-cell clones specific for p21 ras-derived synthetic peptides. Hum Immunol (1992) 33:266–74. 10.1016/0198-8859(92)90334-J 1639630

[29] Fossum B Olsen AC Thorsby E Gaudernack G. CD8+ T cells from a patient with colon carcinoma, specific for a mutant p21-Ras-derived peptide (Gly13–>Asp), are cytotoxic towards a carcinoma cell line harbouring the same mutation. Cancer Immunol Immunother (1995) 40:165–72. 10.1007/BF01517348 PMC11037639 7728775

[30] Gjertsen KM Bakka A Breivik J Saeterdal I. Vaccination with mutant ras peptides and induction of T-cell responsiveness in pancreatic carcinoma patients carrying the corresponding RAS mutation. Lancet (1995) 346:1399–400. 10.1016/s0140-6736(95)92408-6 7475823

[31] Rahma OE Hamilton JM Wojtowicz M Dakheel O Bernstein S Liewehr DJ. The immunological and clinical effects of mutated ras peptide vaccine in combination with IL-2, GM-CSF, or both in patients with solid tumors. J Transl Med (2014) 12:1–12. 10.1186/1479-5876-12-55 24565030 PMC3942063

[32] Toubaji A Achtar M Provenzano M Herrin VE Behrens R Hamilton M. Pilot study of mutant ras peptide-based vaccine as an adjuvant treatment in pancreatic and colorectal cancers. Cancer Immunol Immunother (2008) 57:1413–20. 10.1007/s00262-008-0477-6 PMC11030622 18297281

[33] Wedén S Klemp M Gladhaug IP Mãller M Eriksen JA Gaudernack G. Long-term follow-up of patients with resected pancreatic cancer following vaccination against mutant K-ras. Int J Cancer (2011) 128:1120–8. 10.1002/ijc.25449 20473937

[34] Tefferi A Cervantes F Mesa R Passamonti F Verstovsek S Vannucchi AM. Revised response criteria for myelofibrosis: International Working Group-Myeloproliferative Neoplasms Research and Treatment (IWG-MRT) and European LeukemiaNet (ELN) consensus report. Blood (2013) 122:1395–8. 10.1182/blood-2013-03-488098 PMC4828070 23838352

[35] Barosi G Mesa R Finazzi G Harrison C Kiladjian JJ Lengfelder E. Revised response criteria for polycythemia vera and essential thrombocythemia: An ELN and IWG-MRT consensus project. Blood (2013) 121:4778–81. 10.1182/blood-2013-01-478891 PMC3674675 23591792

[36] de Vries IJM Bernsen MR Lesterhuis WJ Scharenborg NM Strijk SP Gerritsen M-JP. Immunomonitoring tumor-specific T cells in delayed-type hypersensitivity skin biopsies after dendritic cell vaccination correlates with clinical outcome. J Clin Oncol (2005) 23:5779–87. 10.1200/JCO.2005.06.478 16110035

[37] Holmström MO Andersen MH. Healthy donors harbor memory T cell responses to RAS neo-antigens. Cancers (Basel) (2020) 12:3045. 10.3390/cancers12103045 PMC7589254 33086698

[38] Moodie Z Price L Gouttefangeas C Mander A Janetzki S Löwer M. Response definition criteria for ELISPOT assays revisited. Cancer Immunol Immunother (2010) 59:1489–501. 10.1007/s00262-010-0875-4 PMC2909425 20549207

[39] Jørgensen NG Klausen U Grauslund JH Helleberg C Aagaard TG Do TH. Peptide Vaccination Against PD-L1 With IO103 a Novel Immune Modulatory Vaccine in Multiple Myeloma : A Phase I First-in-Human Trial. Front Immunol (2020) 11:1–11. 10.3389/fimmu.2020.595035 33240282 PMC7680803

[40] Wang JC Chen C Kundra A Kodali S Pandey A Wong C. Programmed Cell Death Receptor (PD-1) Ligand (PD-L1) expression in Philadelphia chromosome-negative myeloproliferative neoplasms. Leuk Res (2019) 79:52–9. 10.1016/j.leukres.2019.02.010 30851544

[41] Bozkus CC Roudko V Finnigan JP Mascarenhas J Hoffman R Iancu-Rubin C. Immune checkpoint blockade enhances shared neoantigen-induced T-cell immunity directed against mutated calreticulin in myeloproliferative neoplasms. Cancer Discov (2019) 9:1192–207. 10.1158/2159-8290.CD-18-1356 PMC6726533 31266769

[42] Schumacher TN Schreiber RD. Neoantigens in cancer immunotherapy. Science (2015) 348:69–74. 10.1126/science.aaa4971 25838375

[43] Khleif SN Abrams SI Hamilton JM Bergmann-Leitner E Chen A Bastian A. A phase I vaccine trial with peptides reflecting ras oncogene mutations of solid tumors. J Immunother (1999) 22:155–65. 10.1097/00002371-199903000-00007 10093040

[44] Griffiths EA Srivastava P Matsuzaki J Brumberger Z Wang ES Kocent J. NY-ESO-1 Vaccination in Combination with Decitabine Induces Antigen-Specific T-Lymphocyte Responses in Patients with Myelodysplastic Syndrome. Clin Cancer Res (2017) 24:1019–29. 10.1158/1078-0432.CCR-17-1792 PMC5844797 28947565

[45] Maslak PG Dao T Bernal Y Chanel SM Zhang R Frattini M. Phase 2 trial of a multivalent WT1 peptide vaccine (galinpepimut-S) in acute myeloid leukemia. Blood Adv (2018) 2:224–34. 10.1182/bloodadvances.2017014175 PMC5812332 29386195

[46] Greiner J Ono Y Hofmann S Schmitt A Mehring E Götz M. Mutated regions of nucleophosmin 1 elicit both CD4+ and CD8+ T-cell responses in patients with acute myeloid leukemia. Blood (2016) 97:1282–90. 10.1182/blood-2011-11-394395.There 22592607

[47] Greiner J Schneider V Schmitt M Götz M Döhner K Wiesneth M. Immune responses against the mutated region of cytoplasmatic NPM1 might contribute to the favorable clinical outcome of AML patients with NPM1 mutations (NPM1mut). Blood (2013) 122:1087–8. 10.1182/blood-2013-04-496844 23929838

[48] Balachandran VP Luksza M Zhao JN Makarov V Moral JA Remark R. Identification of unique neoantigen qualities in long-term survivors of pancreatic cancer. Nature (2017) 551:S12–6. 10.1038/nature24462 PMC6145146 29132146

[49] Rahma OE Gammoh E Simon RM Khleif SN. Is the “3+3” dose-escalation Phase i Clinical trial design suitable for therapeutic cancer vaccine development? A recommendation for alternative design. Clin Cancer Res (2014) 20:4758–67. 10.1158/1078-0432.CCR-13-2671 PMC4167476 25037736

[50] Elf S Abdelfattah NS Chen E Perales-Pat??n J Rosen EA Ko A. Mutant calreticulin requires both its mutant C-terminus and the thrombopoietin receptor for oncogenic transformation. Cancer Discov (2016) 6:368–81. 10.1158/2159-8290.CD-15-1434 PMC4851866 26951227

[51] Nivarthi H Chen D Cleary C Kubesova B Jäger R Bogner E. Thrombopoietin receptor is required for the oncogenic function of CALR mutants. Leukemia (2016) 30:1759–63. 10.1038/leu.2016.32 PMC4980558 26883579

[52] Chachoua I Pecquet C El-Khoury M Nivarthi H Albu RI Marty C. Thrombopoietin receptor activation by myeloproliferative neoplasm associated calreticulin mutants. Blood (2016) 127:1325–35. 10.1182/blood-2015-11-681932 26668133

[53] Araki M Yang Y Masubuchi N Hironaka Y Takei H Morishita S. Activation of the thrombopoietin receptor by mutant calreticulin in CALR-mutant myeloproliferative neoplasms. Blood (2016) 127:1307–16. 10.1182/blood-2015-09-671172 26817954

[54] Tefferi A Guglielmelli P Larson DR Finke C Wassie EA Pieri L. Long-term survival and blast transformation in molecularly annotated essential thrombocythemia, polycythemia vera, and myelofibrosis. Blood (2014) 124:2507–13. 10.1182/blood-2014-05-579136 PMC4199952 25037629

[55] Rumi E Pietra D Pascutto C Guglielmelli P Martínez-Trillos A Casetti I. Clinical effect of driver mutations of JAK2, CALR, or MPL in primary myelofibrosis. Blood (2014) 124:1062–9. 10.1182/blood-2014-05-578435 PMC4133481 24986690

[56] Qazilbash MH Wieder E Thall PF Wang X Rios R Lu S. PR1 peptide vaccine induces specific immunity with clinical responses in myeloid malignancies. Leukemia (2017) 31:697–704. 10.1038/leu.2016.254 27654852 PMC5332281

[57] Kenter GG Welters MJP Valentijn a RPM Lowik MJG Berends-van der Meer D Vloon APG. Vaccination against HPV-16 oncoproteins for vulvar intraepithelial neoplasia. N Engl J Med (2009) 361:1838–47. 10.1056/NEJMoa0810097 19890126

[58] Holmström MO Riley CH Skov V Svane IM Hasselbalch HC Andersen MH. Spontaneous T-cell responses against the immune check point programmed-death-ligand 1 (PD-L1) in patients with chronic myeloproliferative neoplasms correlate with disease stage and clinical response. Oncoimmunology (2018) 7:e1433521. 10.1080/2162402X.2018.1433521 29872567 PMC5980374

[59] Aaboe-Jørgensen M Holmström MO Martinenaite E Riley CH Hasselbalch HC Hald Andersen M. Spontaneous T-cell responses against Arginase-1 in chronic myeloproliferative neoplasms relative to disease stage and type of driver mutation. Oncoimmunology (2018) 7(9):e1468957. 10.1080/2162402X.2018.1468957 30228936 PMC6140593

[60] Romano M Sollazzo D Trabanelli S Barone M Polverelli N Perricone M. Mutations in JAK2 and Calreticulin genes are associated with specific alterations of the immune system in myelofibrosis. Oncoimmunology (2017) 6:e1345402. 10.1080/2162402X.2017.1345402 29123956 PMC5665081

[61] Holmström MO Ahmad SM Klausen U Bendtsen SK Martinenaite E Riley CH. High frequencies of circulating memory T cells specific for calreticulin exon 9 mutations in healthy individuals. Blood Cancer J (2019) 9:8. 10.1038/s41408-018-0166-4 30655510 PMC6336769

[62] Blankenstein T Coulie PG Gilboa E Jaffee EM. The determinants of tumour immunogenicity. Nat Rev Cancer (2012) 12:307–13. 10.1038/nrc3246 PMC3552609 22378190

[63] Arshad N Cresswell P. Tumor-associated calreticulin variants functionally compromise the peptide loading complex and impair its recruitment of MHC-I. J Biol Chem (2018) 293:9555–69. 10.1074/jbc.RA118.002836 PMC6016473 29769311

[64] Aarntzen EHJG Bol K Schreibelt G Jacobs JFM Lesterhuis WJ Van Rossum MM. Skin-test infiltrating lymphocytes early predict clinical outcome of dendritic cell-based vaccination in metastatic melanoma. Cancer Res (2012) 72:6102–10. 10.1158/0008-5472.CAN-12-2479 23010076

[65] Westdorp H Creemers JHA Van Oort IM Schreibelt G Gorris MAJ Mehra N. Blood-derived dendritic cell vaccinations induce immune responses that correlate with clinical outcome in patients with chemo-naive castration-resistant prostate cancer. J Immunother Cancer (2019) 7:1–15. 10.1186/s40425-019-0787-6 31727154 PMC6854814

[66] Cai Y Fleming C Yan J. New insights of T cells in the pathogenesis of psoriasis. Cell Mol Immunol (2012) 9:302–9. 10.1038/cmi.2012.15 PMC4132586 22705915

[67] Kristinsson SY Landgren O Samuelsson J Björkholm M Goldin LR. Autoimmunity and the risk of myeloproliferative neoplasms. Haematologica (2010) 95:1216–20. 10.3324/haematol.2009.020412 PMC2895049 20053870

[68] Kjaer L Holmström MO Cordua S Andersen MH Svane IM Thomassen M. Sorted Peripheral Blood Cells Identify CALR Mutations in B- and T-lymphocytes. Leuk Lymphoma (2017) 59:973–7. 10.1080/10428194.2017.1359743 28792253

[69] Sallusto F Geginat J Lanzavecchia A. Central memory and effector memory T cell subsets: Function, generation, and maintenance. Annu Rev Immunol (2004) 22:745–63. 10.1146/annurev.immunol.22.012703.104702 15032595

[70] Sallusto F Lenig D Förster R Lipp M Lanzavecchia A. Two subsets of memory T lymphocytes with distinct homing potentials and effector functions. Nature (1999) 401:708–12. 10.1038/44385 10537110

[71] Riley CH Jensen MK Brimnes MK Hasselbalch HC Bjerrum OW Straten PT. Increase in circulating CD4+CD25+Foxp3+ T cells in patients with Philadelphia-negative chronic myeloproliferative neoplasms during treatment with IFN-α. Blood (2011) 118:2170–3. 10.1182/blood-2011-03-340992 21708889

[72] Riley CH Hansen M Brimnes MK Hasselbalch HC Bjerrum OW Straten PT. Expansion of circulating CD56(bright) natural killer cells in patients with JAK2-positive chronic myeloproliferative neoplasms during treatment with interferon-α. Eur J Haematol (2015) 94:227–34. 10.1111/ejh.12420 25082025

[73] Talmadge JE Gabrilovich DI. History of myeloid-derived suppressor cells. Nat Rev Cancer (2013) 13:739–52. 10.1038/nrc3581 PMC4358792 24060865

[74] Wang JC Kundra A Andrei M Baptiste S Chen C Wong C. Myeloid-derived suppressor cells in patients with myeloproliferative neoplasm. Leuk Res (2016) 43:39–43. 10.1016/j.leukres.2016.02.004 26943702

[75] Han L Schubert C Köhler J Schemionek M Isfort S Brümmendorf TH. Calreticulin-mutant proteins induce megakaryocytic signaling to transform hematopoietic cells and undergo accelerated degradation and Golgi-mediated secretion. J Hematol Oncol (2016) 9:45. 10.1186/s13045-016-0275-0 27177927 PMC4894373

[76] Liu P Zhao L Loos F Marty C Xie W Martins I. Immunosuppression by Mutated Calreticulin Released from Malignant Cells. Mol Cell (2020) 77:1–13. 10.1016/j.molcel.2019.11.004 31785928

[77] Sollazzo D Forte D Polverelli N Perricone M Romano M Luatti S. Circulating Calreticulin Is Increased in Myelofibrosis: Correlation with Interleukin-6 Plasma Levels, Bone Marrow Fibrosis, and Splenomegaly. Mediators Inflamm (2016) 2016:5860657. 10.1155/2016/5860657 27672242 PMC5031875

[78] Skov V Riley CH Thomassen M Kjær L Stauffer Larsen T Bjerrum OW. The impact of interferon-alpha2 on HLA genes in patients with polycythemia vera and related neoplasms. Leuk Lymphoma (2017) 58:1914–21. 10.1080/10428194.2016.1262032 27911124

[79] Lesokhin AM Ansell SM Armand P Scott EC Halwani A Gutierrez M. Nivolumab in Patients With Relapsed or Refractory Hematologic Malignancy: Preliminary Results of a Phase Ib Study. J Clin Oncol (2016) 34:2698–704. 10.1200/JCO.2015.65.9789 PMC5019749 27269947

[80] Panagiota V Thol F Markus B Fehse B Alchalby H Badbaran A. Prognostic effect of calreticulin mutations in patients with myelofibrosis after allogeneic hematopoietic stem cell transplantation. Leukemia (2014) 28:1552–5. 10.1038/leu.2014.66 24504025

[81] Klyuchnikov E Holler E Bornhäuser M Kobbe G Nagler A Shimoni A. Donor lymphocyte infusions and second transplantation as salvage treatment for relapsed myelofibrosis after reduced-intensity allografting. Br J Haematol (2012) 159:172–81. 10.1111/bjh.12013 22909192

[82] Hofmann S Götz M Scheider V Guillaume P Bunjes D Döhner H. Donor Lymphocyte Infusion Induces Polyspecific CD8+ T-Cell Responses With Concurrent Molecular Remission in Acute Myeloid Leukemia With NPM1 Mutation. J Clin Oncol (2013) 31:2013–5. 10.1200/JCO.2012.48.6183 23248243

[83] Verstovsek S Mesa Ra Gotlib J Levy RS Gupta V DiPersio JF. A double-blind, placebo-controlled trial of ruxolitinib for myelofibrosis. N Engl J Med (2012) 366:799–807. 10.1056/NEJMoa1110557 22375971 PMC4822164

[84] Heine A Andrea S Held E Daecke SN Wallner S Yajnanarayana SP. The JAK-inhibitor ruxolitinib impairs dendritic cell function in vitro and in vivo. Blood (2013) 122:1192–202. 10.1182/blood-2013-03-484642 23770777

[85] Schönberg K Rudolph J Vonnahme M Yajnanarayana SP Cornez I Hejazi M. JAK inhibition impairs NK cell function in myeloproliferative neoplasms. Cancer Res (2015) 75:2187–99. 10.1158/0008-5472.CAN-14-3198 25832652

[86] Parampalli Yajnanarayana S Stübig T Cornez I Alchalby H Schönberg K Rudolph J. JAK1/2 inhibition impairs T cell function in vitro and in patients with myeloproliferative neoplasms. Br J Haematol (2015) 169:824–33. 10.1021/ac901991x 25824483

[87] Andersen MH. Anti-regulatory T cells. Semin Immunopathol (2017) 39:317–26. 10.1007/s00281-016-0593-x 27677755

[88] Munir S Andersen GH Met Ö Donia M Frøsig TM Larsen SK. HLA-restricted CTL that are specific for the immune checkpoint ligand PD-L1 occur with high frequency in cancer patients. Cancer Res (2013) 73:1764–76. 10.1158/0008-5472.CAN-12-3507 23328583

[89] Ahmad SM Martinenaite E Holmström M Jørgensen MA Met Ö Nastasi C. The inhibitory checkpoint, PD-L2, is a target for effector T cells: Novel possibilities for immune therapy. Oncoimmunology (2017) 7(2):e1390641. 10.1080/2162402X.2017.1390641 29308318 PMC5749669

[90] Martinenaite E Mortensen REJ Hansen M Orebo Holmström M Munir Ahmad S Grønne Dahlager Jørgensen N. Frequent adaptive immune responses against arginase-1. Oncoimmunology (2017) 7:e1404215. 10.1080/2162402X.2017.1404215 29399404 PMC5790367

[91] Weis-Banke SE Hübbe ML Holmström MO Jørgensen MA Bendtsen SK Martinenaite E. The metabolic enzyme arginase-2 is a potential target for novel immune modulatory vaccines. Oncoimmunology (2020) 9. 10.1080/2162402X.2020.1771142 PMC7458644 32923127

[92] Munir S Larsen SK Iversen TZ Donia M Klausen TW Svane IM. Natural CD4 + T-cell responses against indoleamine 2,3-dioxygenase. PLoS One (2012) 7:e34568. 10.1371/journal.pone.0034568 22539948 PMC3335144

[93] Iversen TZ Engell-Noerregaard L Ellebaek E Andersen R Larsen SK Bjoern J. Long-lasting disease stabilization in the absence of toxicity in metastatic lung cancer patients vaccinated with an epitope derived from indoleamine 2,3 dioxygenase. Clin Cancer Res (2014) 20:221–32. 10.1158/1078-0432.CCR-13-1560 24218513

[94] Svane I-M Kjeldsen JW Lorentzen CL Martinenaite E Andersen MH. LBA48 Clinical efficacy and immunity of combination therapy with nivolumab and IDO/PD-L1 peptide vaccine in patients with metastatic melanoma: A phase I/II trial. Ann Oncol (2020) 31:S1176. 10.1016/j.annonc.2020.08.2278

[95] Andersen MH. The T-win® technology: immune-modulating vaccines. Semin Immunopathol (2019) 41:87–95. 10.1007/s00281-018-0695-8 29968045

[96] Cordua S Kjaer L Skov V Pallisgaard N Hasselbalch HC Ellervik C. Prevalence and phenotypes of JAK2 V617F and Calreticulin mutations in a Danish general population. Blood (2019) 134:469–79. 10.1182/blood.2019001113 31217187

[97] Holmström MO Cordua S Skov V Kjær L Pallisgaard N Ellervik C. Evidence of immune elimination, immuno-editing and immune escape in patients with hematological cancer. Cancer Immunol Immunother (2020) 69:315–24. 10.1007/s00262-019-02473-y PMC11027882 31915854

