# Supplementary figures and images for: Therapeutic Cancer Vaccination With a Peptide Derived From the Calreticulin Exon 9 Mutations Induces Strong Cellular Immune Responses in Patients With CALR-Mutant Chronic Myeloproliferative Neoplasms
Source: Front Oncol. 2021 Feb 26;11:637420. doi: 10.3389/fonc.2021.637420 (PMC7952976; doi:10.3389/fonc.2021.637420)

## Slide 1
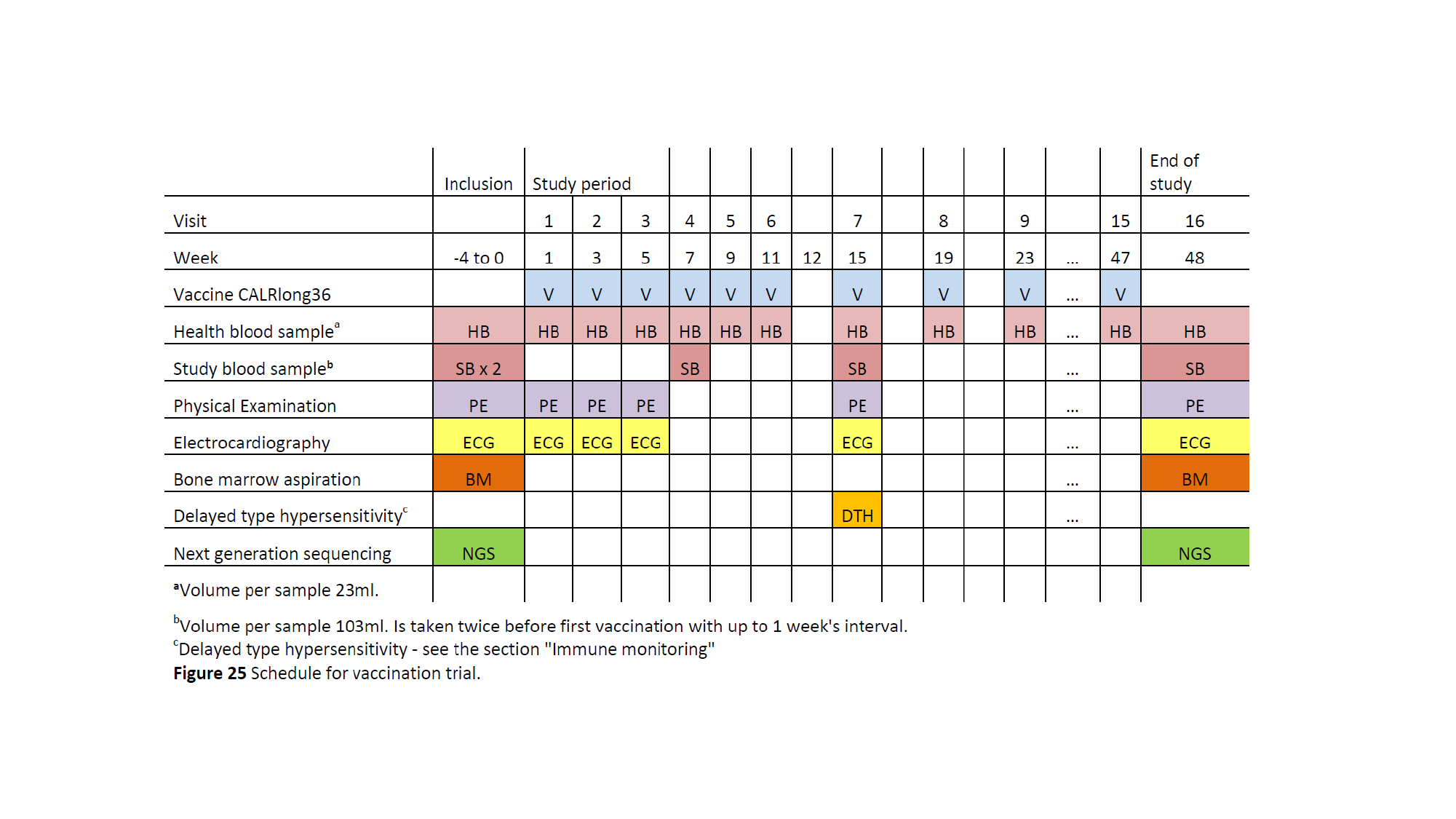

Supplement: Supplementary Figure 1 — Detailed in- and exclusion criteria. [file Presentation_1.pptx]

## Slide 1
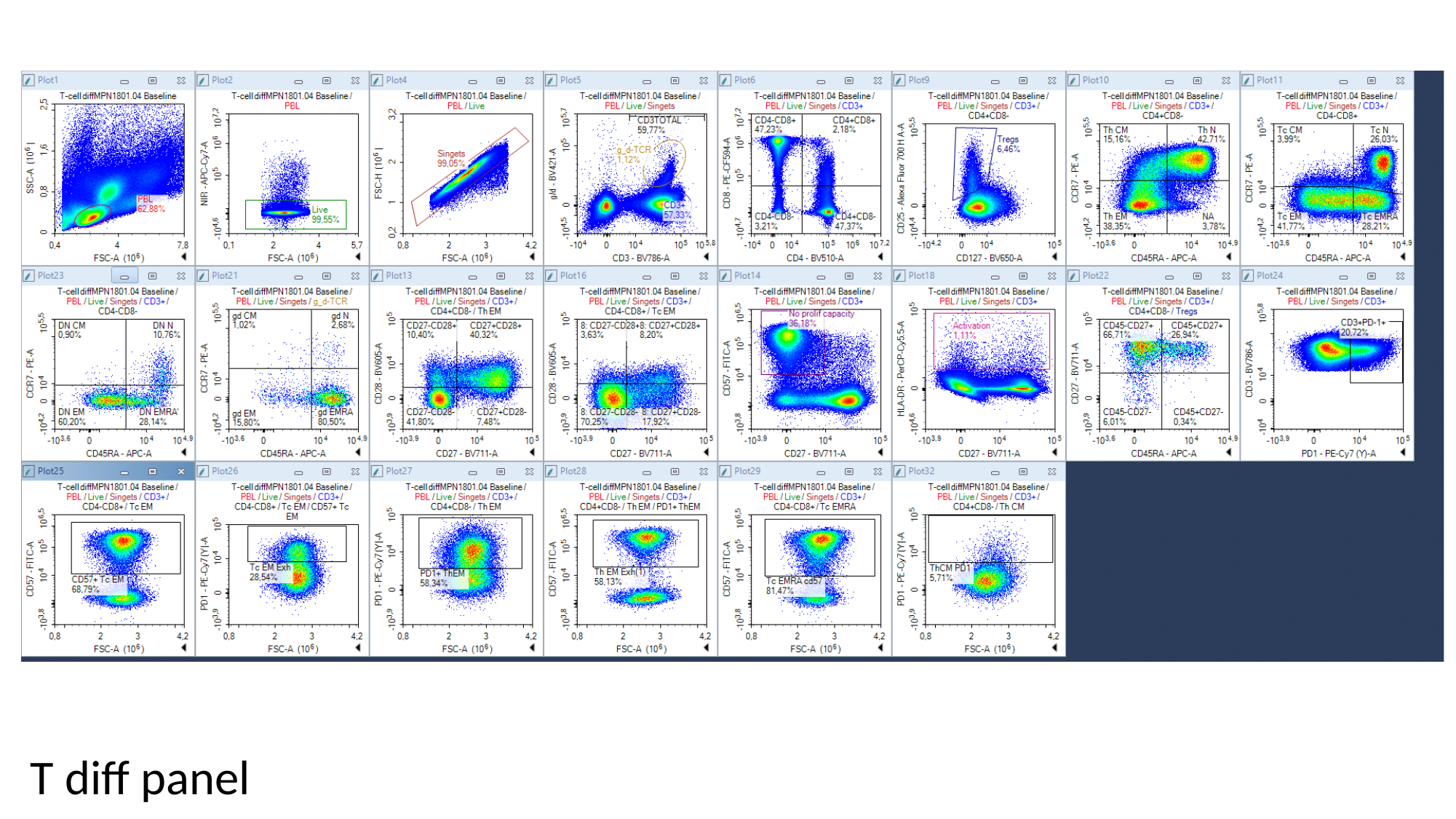

#
T diff panel

## Slide 2
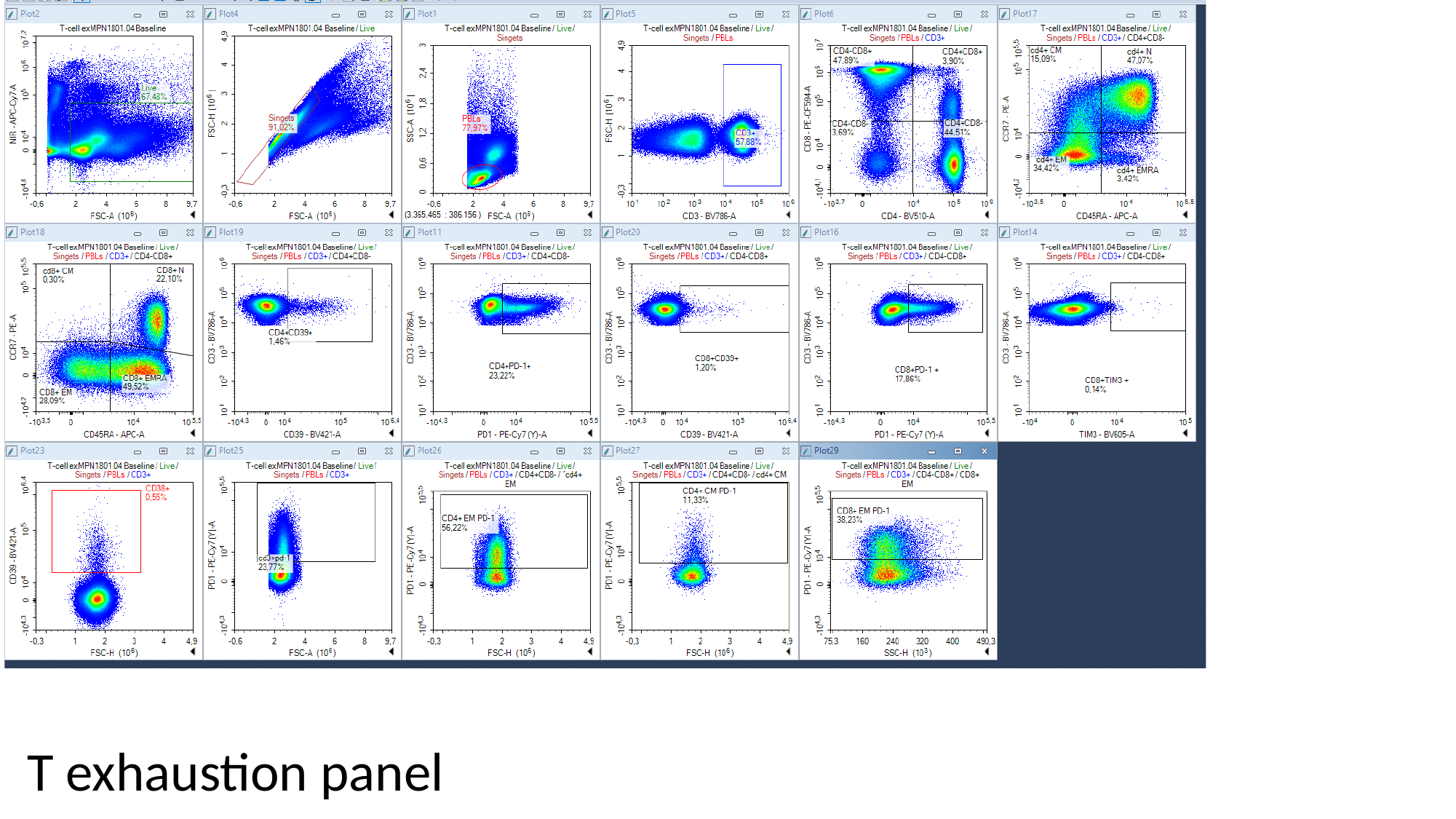

#
T exhaustion panel

## Slide 3
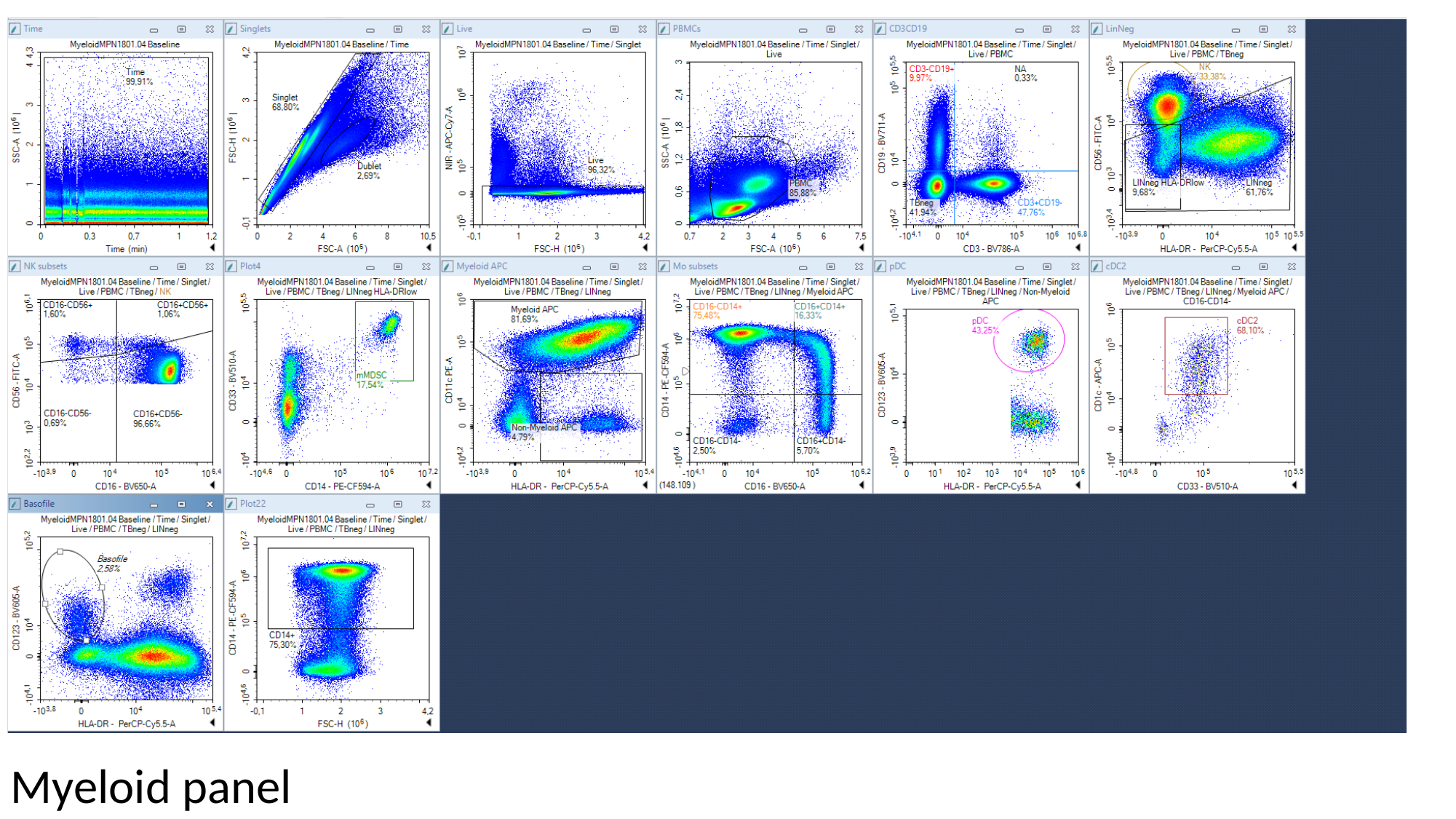

#
Myeloid panel

Supplement: Supplementary Figure 2 — Gant chart showing the vaccination schedule and additional examination/analyses. Abbreviations: V=vaccine, HB=health blood sample, SB=study blood sample, PE=physical examination, ECG=electrocardiogram, BM=bone marrow examination, DTH=delayed type hypersensitivity, NGS=next generation sequencing [file Presentation_2.pptx]

## Slide 1
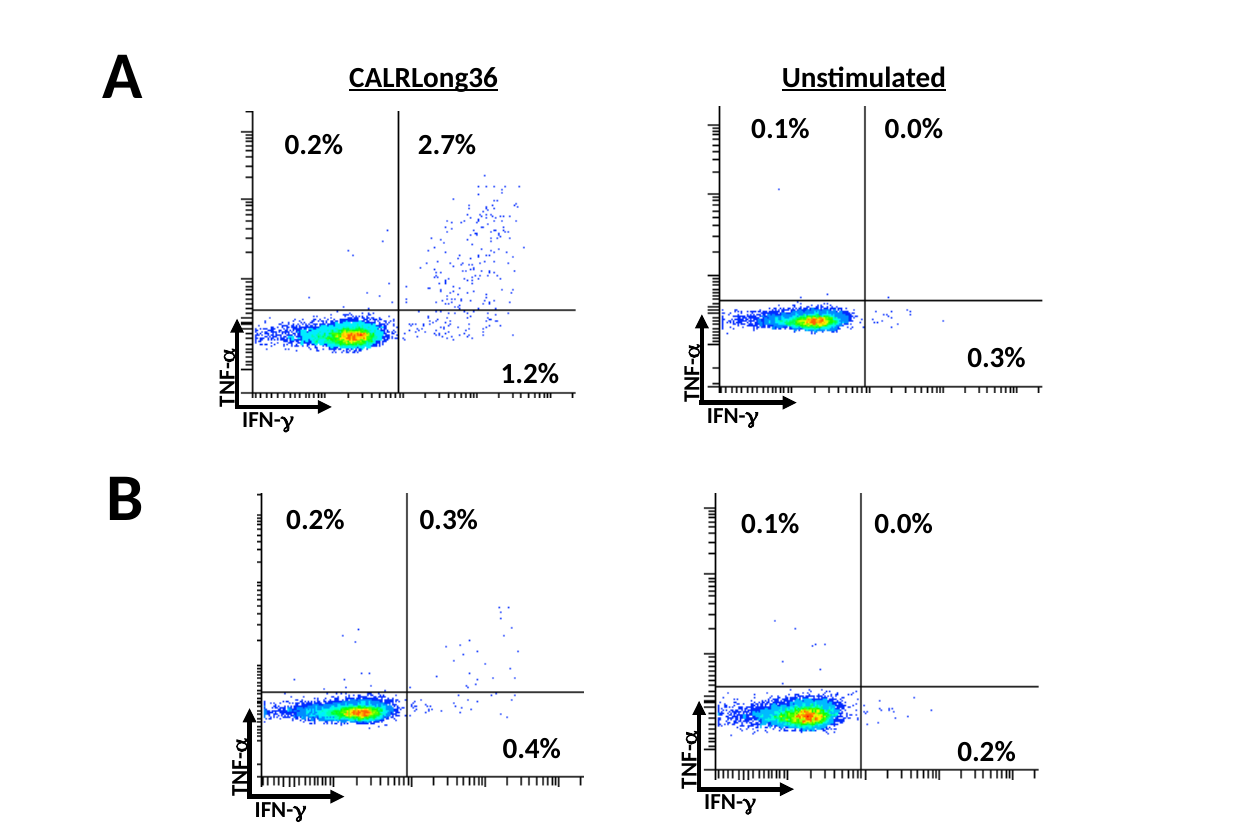

A
CALRLong36
Unstimulated
0.1%
0.0%
0.2%
2.7%
0.3%
TNF-a
TNF-a
1.2%
IFN-g
IFN-g
B
0.2%
0.3%
0.1%
0.0%
TNF-a
0.4%
0.2%
TNF-a
IFN-g
IFN-g

Supplement: Supplementary Figure 5 — Sustained CD8+ T-cell response in patient 2 even after cessation of vaccination, identified by intracellular cytokine staining. (A) CD8+ T-cell response in patient PBMC at 37 weeks after cessation of therapy. (B) CD8+ T-cell response in patient PBMC at 49 weeks after cessation of therapy. [file Presentation_3.pptx]

## Slide 1
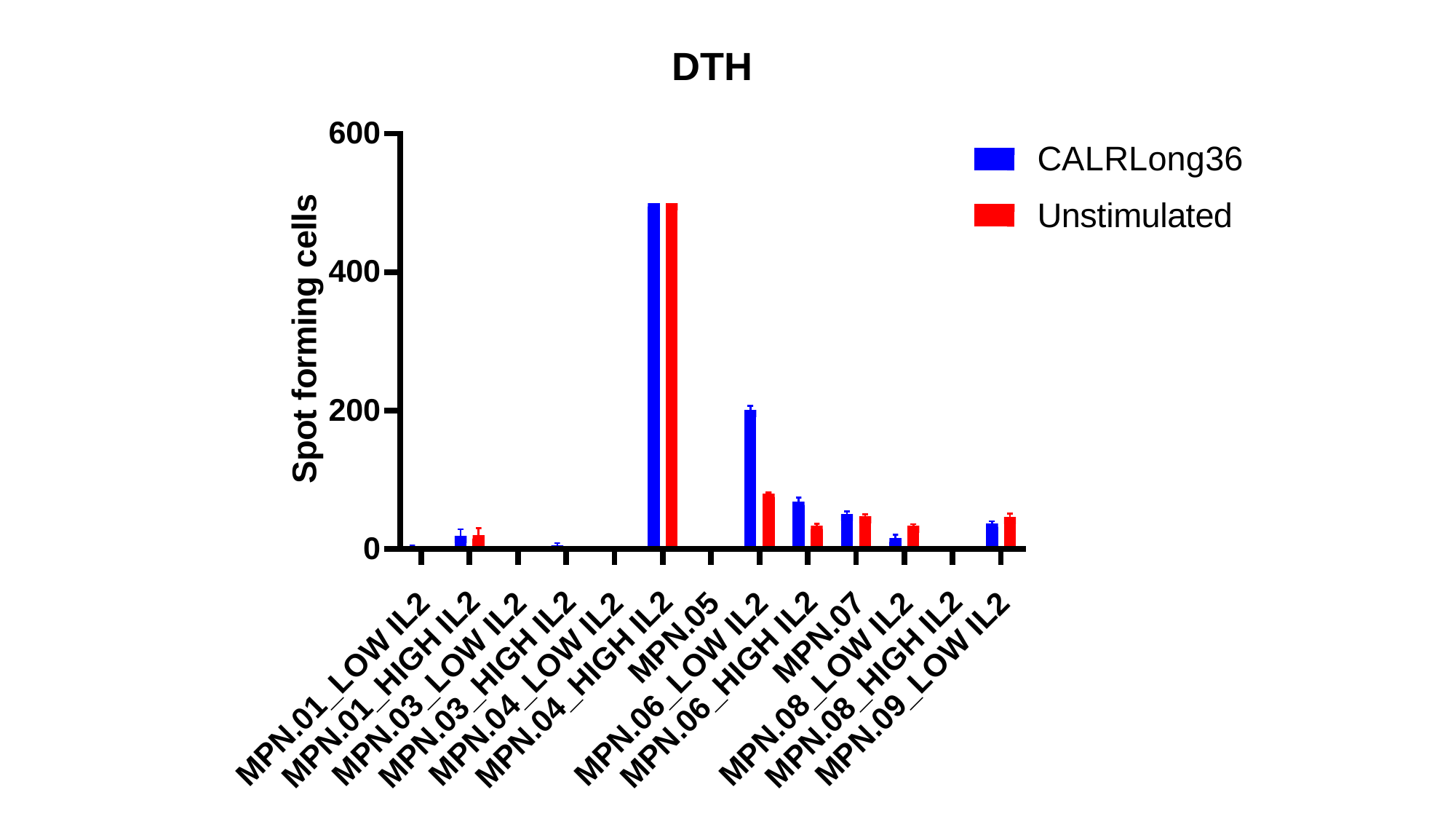

Supplement: Supplementary Figure 6 — Response in skin-infiltrating lymphocytes showing response in both the CALRLong36-stimulated wells and the negative control wells. [file Presentation_4.pptx]
