# Supplementary material for: Therapeutic Cancer Vaccination With a Peptide Derived From the Calreticulin Exon 9 Mutations Induces Strong Cellular Immune Responses in Patients With CALR-Mutant Chronic Myeloproliferative Neoplasms
Source: Front Oncol. 2021 Feb 26;11:637420. doi: 10.3389/fonc.2021.637420 (PMC7952976; doi:10.3389/fonc.2021.637420)
Supplement: Supplementary file 5 [file Table_1.docx]

## Inclusion criteria

The patients must fulfill the following criteria:

1. Diagnosis of essential thrombocythemia, post essential thrombocythemia myelofibrosis, prefibrotic myelofibrosis or primary myelofibrosis according to the WHO criteria.
2. Verified mutation in *CALR* exon 9.
3. Age ≥18 years
4. Performance status ≤ 2 (ECOG-scale)
5. Expected survival > 3 months
6. Sufficient bone marrow function, i.e.
   1. Leucocytes ≥ 1,5 x 10^9^
   2. Granulocytes ≥ 1,0 x 10^9^
   3. Thrombocytes ≥ 20 x 10^9^
   4. Hemoglobin ≥ 7 mmol/L
7. Creatinine < 2.5 upper normal limit, i.e. < 300 µmol/l
8. Sufficient liver function, i.e.
   1. ALAT < 2.5 upper normal limit, i.e. ALAT <112 U/l
   2. Bilirubin < 30 U/l
9. For women: Agreement to use contraceptive methods with a failure rate of < 1% per year during the treatment period and for at least 120 days after the last treatment.
10. For men: Agreement to use contraceptive measures and agreement to refrain from donating sperm.

## Exclusion criteria

1. Other malignancies in the medical history excluding squamous cell carcinoma. Patients cured for another malignant disease with no sign of relapse three years after ended treatment is allowed to enter the protocol.
2. Significant medical condition per investigators judgement e.g. severe Asthma/COPD, poorly regulated heart condition, insulin dependent diabetes mellitus.
3. Acute or chronic viral or bacterial infection e.g. HIV, hepatitis or tuberculosis
4. Serious known allergies or earlier anaphylactic reactions.
5. Known sensibility to Montanide ISA-51
6. Any active autoimmune diseases e.g. autoimmune neutropenia, thrombocytopenia or hemolytic anemia, systemic lupus erythematosus, scleroderma, myasthenia gravis, autoimmune glomerulonephritis, autoimmune adrenal deficiency, autoimmune thyroiditis etc.
7. Pregnant and breastfeeding women.
8. Fertile women not using secure contraception with a failure rate less than < 1%
9. Patients taking immune suppressive medications incl. corticosteroids and methotrexate at the time of enrollment
10. Psychiatric disorders that per investigator judgment could influence compliance.
11. Treatment with other experimental drugs
12. Treatment with other anti-cancer drugs – except IFN-a, hydroxyurea or anagrelide.
13. Treatment with ruxolitinib.
14. Treatment with chemotherapy or immune therapy (excluding IFN-a, hydroxyurea or anagrelide within the last 28 days).
