# Supplementary material for: Therapeutic Cancer Vaccination With a Peptide Derived From the Calreticulin Exon 9 Mutations Induces Strong Cellular Immune Responses in Patients With CALR-Mutant Chronic Myeloproliferative Neoplasms
Source: Front Oncol. 2021 Feb 26;11:637420. doi: 10.3389/fonc.2021.637420 (PMC7952976; doi:10.3389/fonc.2021.637420)
Supplement: Supplementary file 6 [file Table_2.docx]

| **Antigen** | **Fluorochrome** | **Clone** | **Manufacturer** | **Catalog No.** |
| --- | --- | --- | --- | --- |
| TCR γδ | BV421 | B1 | BD Bioscience | 562660 |
| CD39 | BV421 | TU66 | BD Bioscience | 563679 |
| NKG2a | BV421 | 131411 | BD Bioscience | 747924 |
| CD4 | BV510 | SK3 | BD Bioscience | 562970 |
| CD33 | BV510 | WM53 | BD Bioscience | 563257 |
| CD28 | BV605 | L293 | BD Bioscience | 742527 |
| TIM-3 | BV605 | 7D3 | BD Bioscience | 742856 |
| CD123 | BV605 | 7G3 | BD Bioscience | 564197 |
| CD127 | BV650 | A019D5 | Nordic Biosite | 351325 |
| TIGIT | BV650 | 741182 | BD Bioscience | 747840 |
| CD16 | BV650 | 3G8 | BD Bioscience | 563692 |
| CD27 | BV711 | L128 | BD Bioscience | 563167 |
| CD19 | BV711 | SJ25C1 | BD Bioscience | 563038 |
| CD3 | BV786 | SK7 | BD Bioscience | 563800 |
| CD57 | FITC | NK-1 | BD Bioscience | 555619 |
| LAG3 | FITC | 17B4 | LS Bio | LS-B2237 |
| CD56 | FITC | NCAM16.2 | BD Bioscience | 340410 |
| HLA-DR | PerCP-Cy5.5 | G46-6 | BD Bioscience | 560652 |
| DNAM-1 | PerCP-Cy5.5 | 11A8 | Nordic Biosite | 338313 |
| CCR7 | PE | G043H7 | Nordic Biosite | 353204 |
| CD11c | PE | S-HCL-3 | BD Bioscience | 347637 |
| CD8 | PE-CF594 | HIT8α | BD Bioscience | 566860 |
| CD14 | PE-CF594 | MφP9 | BD Bioscience | 562335 |
| PD-1 | PE-Cy7 | EH12.1 | BD Bioscience | 561272 |
| CD45RA | APC | HI100 | BD Bioscience | 550855 |
| CD1c | APC | L161 | Nordic Biosite | 331524 |
| CD25 | APC-R700 | 2A3 | BD Bioscience | 565106 |
| NIR (Live/dead) | APC-Cy7 | - | ThermoFischer^TM^ | L34976 |
